# Endogenous Fluorescence Signatures in Living Pluripotent Stem Cells Change with Loss of Potency

**DOI:** 10.1371/journal.pone.0043708

**Published:** 2012-08-29

**Authors:** Jayne M. Squirrell, Jimmy J. Fong, Carlos A. Ariza, Amber Mael, Kassondra Meyer, Nirupama K. Shevde, Avtar Roopra, Gary E. Lyons, Timothy J. Kamp, Kevin W. Eliceiri, Brenda M. Ogle

**Affiliations:** 1 Laboratory for Optical and Computational Instrumentation, University of Wisconsin, Madison, Wisconsin, United States of America; 2 Biomedical Engineering, University of Wisconsin, Madison, Wisconsin, United States of America; 3 WiCell Research Institute, Madison, Wisconsin, United States of America; 4 Neuroscience, University of Wisconsin, Madison, Wisconsin, United States of America; 5 Cell and Regenerative Biology, University of Wisconsin, Madison, Wisconsin, United States of America; 6 Medicine, University of Wisconsin, Madison, Wisconsin, United States of America; 7 Material Science Program, University of Wisconsin, Madison, Wisconsin, United States of America; National University of Singapore, Singapore

## Abstract

The therapeutic potential of stem cells is limited by the non-uniformity of their phenotypic state. Thus it would be advantageous to noninvasively monitor stem cell status. Driven by this challenge, we employed multidimensional multiphoton microscopy to quantify changes in endogenous fluorescence occurring with pluripotent stem cell differentiation. We found that global and cellular-scale fluorescence lifetime of human embryonic stem cells (hESC) and murine embryonic stem cells (mESC) consistently decreased with differentiation. Less consistent were trends in endogenous fluorescence intensity with differentiation, suggesting intensity is more readily impacted by nuances of species and scale of analysis. What emerges is a practical and accessible approach to evaluate, and ultimately enrich, living stem cell populations based on changes in metabolism that could be exploited for both research and clinical applications.

## Introduction

The developmental plasticity of stem cells is, simultaneously, the greatest benefit and a major challenge for utilizing stem cells for regenerative therapies. This plasticity is a challenge because we do not understand the myriad of cues capable of directing differentiation. This is particularly problematic with pluripotent stem cells whose ability to generate cells of all embryonic germ layers presents the very real problem of teratoma formation when these cells are used for tissue repair [1], [2]. To circumvent this problem, one method would be to separate the differentiating cells from those that retain pluripotency. Current strategies toward this end usually incorporate either a lineage specific selectable marker, such as antibiotic resistance, lineage specific fluorescent tags or cell surface fluorescent antibodies, to be used in conjunction with fluorescence activated cell sorting (FACS) [3], to identify and select the cell population of choice. Unfortunately, these techniques have limited clinical applicability because they require invasive manipulation of the cells. Most current cell sorting techniques have the additional disadvantage that cells must be dissociated from each other prior to sorting. Three dimensional cell interactions are important in stem cell differentiation and changing these interactions can alter the developmental progression of the cells [4]. Although techniques are being developed to count and potentially sort 3D cellular entities [5]–[7], such as stem cell aggregates, the use of extrinsic labels is still necessary. The utility of stem cells, both clinically and scientifically, would be enhanced by the identification of non-invasive, intrinsic indicators of developmental status, including viability, differentiation state, and cell lineage.

One intrinsic indicator of cell state is endogenous fluorescence. Fluorescence has multiple properties that could potentially be used to generate a signature of cell state. Three of those properties can be identified with current microscopy technologies: excitation and emission spectra, which are molecule dependent; intensity, which is concentration dependent; and lifetime, or the time which the electron of a fluorophore stays in the excited state before returning to the ground state. This fluorescence lifetime parameter is concentration independent but context dependent and can vary with environmental parameters such as pH, oxygen, and binding partners [8].

A variety of biological molecules exhibit fluorescence including collagen, retinol, folic acid, pyridoxine, tyrosine, lipofuscin, tryptophan and melanin [9]–[13]. Of particular interest are metabolic cofactors, such as nicotinamide adenine dinucleotide (NADH) and flavin adenine dinucleotide (FAD). Alterations in metabolic profiles occurring with changes in cell status could be identified by examining the fluorescence of these metabolites. Additionally, these compounds are relatively abundant in cells, particularly NADH, compared to other endogenous fluorophores [14], making them more readily accessible to interrogation. The pyridine nucleotides, NADH and NADPH, have an excitation peak at 340 nm [15] and are amenable to examination with multiphoton excitation [16]. Although NADH and NADPH cannot be separated using these optical methods [17], the majority of the fluorescence is likely to be from NADH [18]. Therefore in this paper we refer to the endogenous fluorescence as a reflection of NADH, with the understanding that this fluorescence may include small quantities of other fluorophores. Several studies have shown that NADH fluorescence is indicative of changes in the metabolic status of cells. NADH fluorescence has been used to monitor metabolic responses to calcium perturbation [17], cancer progression [19], [20], hypoxia [21], proliferation [22] and cell death [23], [24].

Metabolism is a likely candidate for identifying changes in the state of pluripotent stem cells. Certain changes in metabolism are associated with cell death [23], thus metabolism could be used to assess the viability of cells and cell aggregates, which would be helpful for identifying the healthiest cell clusters to be used for transplantation. Furthermore, a variety of studies suggest that embryonic stem cells (ESCs) are metabolically distinct from differentiated cells. ESCs can proliferate, and may preferentially maintain pluripotency, in a low oxygen environment [25]. Mitochondria in ESCs resemble the mitochondria found in early cleavage stage embryos, tending to be spherical rather than elongated and lacking well-developed cristae [26]. Changes in metabolic proteins and mitochondrial activity have been observed with differentiation of ESCs [27]–[29]. Many of these studies suggest that ESCs, as well as induced pluripotent cells (iPS) [30] preferentially use, or are at least capable of using, non-oxidative pathways and that there is a maturation of mitochondrial metabolism, thus oxidative phosphorylation, with differentiation toward more mature cell types. However, others report that pluripotent cells exhibit a high level of oxidative metabolism [31]. In spite of such discrepancy between studies, the emerging data on ESC and iPS metabolism indicate that pluripotent cells are metabolically special, thus the hypothesis that pluripotent cells could be distinguished from differentiated cells based on metabolic profiling is well founded. Furthermore, metabolic changes may identify sub-populations of differentiating cells as they commit to particular lineages. For example, the intensity of a mitochondrial label can identify cardiomyocytes differentiated from stem cells [32].

Intrinsic fluorescence signatures, fluorescence lifetime in particular, could be used to identify changes in cell status. Cancerous cells exhibit different endogenous fluorescence lifetime profiles than their non-cancerous counterparts [19], [33]–[35]. Fluorescence lifetime analysis has been used to show changes in the ratio of bound to free NADH increases with differentiation of germ cells [36]. Differences in endogenous fluorescence lifetime are detected in adult stem cells as they differentiate. Human mesenchymal stem cells (MSCs) [37]–[39] and salivary gland stem cells [40] show an increase in the fluorescence lifetime of bound NADH as they differentiate and a recent study shows that fluorescence lifetime identifies a decrease in lipid droplet associated granules and an increase in NADH concentration in human embryonic stem cells induced to differentiate toward a neurogenic or trophectoderm lineages [41].

Based on this information, we hypothesized that the endogenous fluorescence profile of embryonic stem cells will change as the cells exit from a pluripotent state and initiate differentiation. It is important to identify these changes on a global level, namely as changes in the endogenous fluorescence parameters of a colony or an aggregate. Pluripotent cells are often cultured as aggregates (embryoid bodies) to promote differentiation, and the three dimensional organization may provide important informational cues that can be lost when the aggregate is dissociated. In parallel, there is merit in examining the changes at the cellular level to better understand the global changes observed. Therefore, to test our hypothesis, we employed a variety of experimental design strategies – distinct time points during spontaneous and forced differentiation, repeated time points to generate timelines of individual aggregate differentiation, and a GFP-tagged marker to track changes in potency on a cellular level - to gather the unique information available from each of the methodologies. Taking advantage of the depth penetration and live cell imaging capabilities of multiphoton laser scanning microscopy (MPLSM) [42], [43], we detected changes in endogenous fluorescence that occur as ESCs, both mouse and human, begin to differentiate. Although the details underlying the metabolic differences that lead to these changes in fluorescence parameters have yet to be determined, this study lays a foundation by characterizing these intrinsic signals and serves as a basis for establishing endogenous fluorescence as a non-invasive indicator of embryonic stem cell differentiation state, which could have a substantial impact on identifying and segregating particular stem cell populations.

## Results

### Global Endogenous Fluorescence Intensity Increased with EB Age

**Figure 1:**
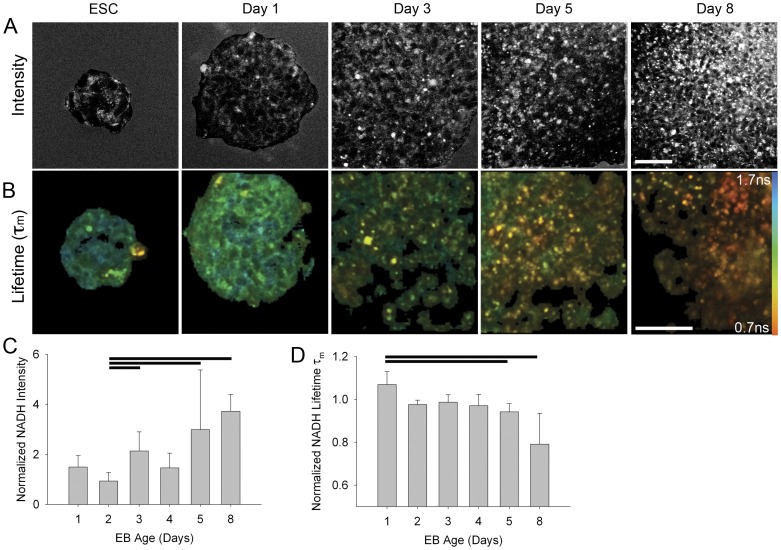
Changes in global endogenous fluorescence intensity fluorescence lifetime with differentiation of mESCs. A, B. MPLSM single optical sections of mouse ESCs at different stages of differentiation (ESC and days 1, 3, 5, and 8 of EB formation; ESC: n = 24, 27, 14, 23, 13, 4 colonies, respectively; EB: n = 7, 16, 6, 18, 8, 4 EBs, respectively) using 780 nm excitation. A. Endogenous fluorescence intensity images following application of a background subtraction FIJI plugin macro (see Methods) to remove 90% of the background of the region of interest prior to intensity analysis. Complete timeline of images, including raw image data, is in Figure S1. B. Color mapped lifetime values of MPLSM single optical sections of mouse ESCs at different stages of differentiation (mESC and days 1, 3, 5, and 8 of EB formation; ESC: n = 13, 20, 8, 18, 10, 18 colonies, respectively; EB: n = 7, 7, 6, 12, 7, 3 EBs respectively) using 780 nm excitation. Color bar indicates lifetime values for τ_m_. Detailed statistics in text and Figure S2. Scale bars = 50µm. C. Background subtracted fluorescence levels were normalized by the mean background subtracted intensity per pixel of the mESCs for that day. For this, and all subsequent figures, bars are mean ± standard deviation. Horizontal lines indicate statistical difference (*P*<0.05) between normalized EB intensity values (Dunn’s method pairwise multiple comparison following ANOVA). D. Quantitation of lifetime values for τ_m_ of NADH (780 nm excitation). Values are normalized to corresponding mean mESC lifetime values. Horizontal lines indicate statistical difference (*P*<0.05) between normalized mEB lifetime values (Dunn’s method pairwise multiple comparison following ANOVA).

Embryonic stem cells (ESCs) can spontaneously differentiate into cells types of the three germ layers following the formation of cellular aggregates termed embryoid bodies (EBs) [44]. To determine whether endogenous fluorescence intensity of NADH changes with differentiation, the global fluorescence intensity level following 780 nm excitation was assessed in mouse embryonic stem cells (mESCs) on days 1, 2, 3, 4, 5 and 8 after mEB formation. For each mEB time point, images of mESC colonies (pluripotent) were also collected to serve as a baseline. To assess changes in intensity over time, we developed a plugin for FIJI ImageJ that permitted a consistent, user-interactive quantitation of fluorescence intensity (see Methods). We set the plugin to subtract 90% of the background value from the pixels in an outlined region of interest. To further adjust for day-to-day variability, the intensity values were normalized to the mean of the mESC intensity for each day. The intensity of mEBs changed with time of differentiation, generally increasing over time (Fig. 1A, C; Fig. S1). This trend was statistically significant (*P*≤0.001; Kruskal-Wallis one way ANOVA on ranks) with the most dramatic difference detected between day 2 and day 8 (*P*<0.05; day 2 normalized intensity = 0.933±0.34, n = 16; day 8 normalized intensity = 3.7±0.68; n = 4; Dunn’s method for multiple pairwise comparison) suggesting that, at the global level, the amount of NADH increases with differentiation of mouse EBs.

### Global Fluorescence Lifetime Decreased with Differentiation

Fluorescence intensity can provide important information about changes in the amount of a fluorophore of interest whereas fluorescence lifetime can identify changes in the microenvironment, as may occur during differentiation. The fluorescence lifetime of a sample can be determined by exponential curve-fitting software that fits the photon counting data to a model function at each pixel to describe the exponential fluorescence decay [45]. In the case of NADH, two components contribute to the fluorescence lifetime decay curve, namely unbound cofactor and protein bound cofactor [8]. The model for the decay curve must include these two contributions, thus the model is: I(t) = α_1_e^−t/τ1^+ α_2_e^−t/τ2^, where I(t) is the intensity decay profile, τ_1_ is the short lifetime component (unbound NADH), τ_2_ is the long lifetime component (bound NADH), α_1_ is the percent contribution of the τ_1_ component and α_2_ is the percent contribution of τ_2_. The mean lifetime τ_m_ was also calculated as τ_m_ = α_1_τ_1_+ α_2_τ_2_. To examine the changing metabolic profiles during differentiation, as reflected by fluorescence lifetime, we generated a developmental timeline. This was accomplished by collecting data on mouse EBs on days 1, 2, 3, 4, 5 and 8 after mEB formation, using 780 nm excitation (NADH). We found that the mean lifetime (τ_m_) of EBs was different across time points generally decreasing as the mEB age increased (*P*≤0.001, Kruskal-Wallis ANOVA), with the normalized τ_m_ of EB day 5 and day 8 being significantly different from the normalized τ_m_ of day 1 mEBs (*P*<0.05, Dunn’s method) (Fig. 1B,D; Fig. S2). When the components that contribute to the τ_m_ were examined, the observed decrease in τ_m_ appeared to be due to a combination of the corresponding decrease τ_2_ lifetime (*P*≤0.001, Kruskal-Wallis ANOVA) (Fig. S2) and a decrease in the percentage of bound NADH (α_2_) (*P*≤0.001; Kruskal-Wallis ANOVA), especially at the later time points. τ_1_ exhibited a similar, though slightly less significant, trend (*P* = 0.015; Kruskal-Wallis ANOVA) (Fig. S2). These changes in fluorescence lifetime may indicate alterations in the state of NADH, possibly changes in binding partners and the ratio of bound NADH to free NADH, suggestive of changes in metabolic profile of the cells.

### Lactate Production Decreases with EB Development and the Efficient Formation of Beating Areas Requires Glucose

**Figure 2:**
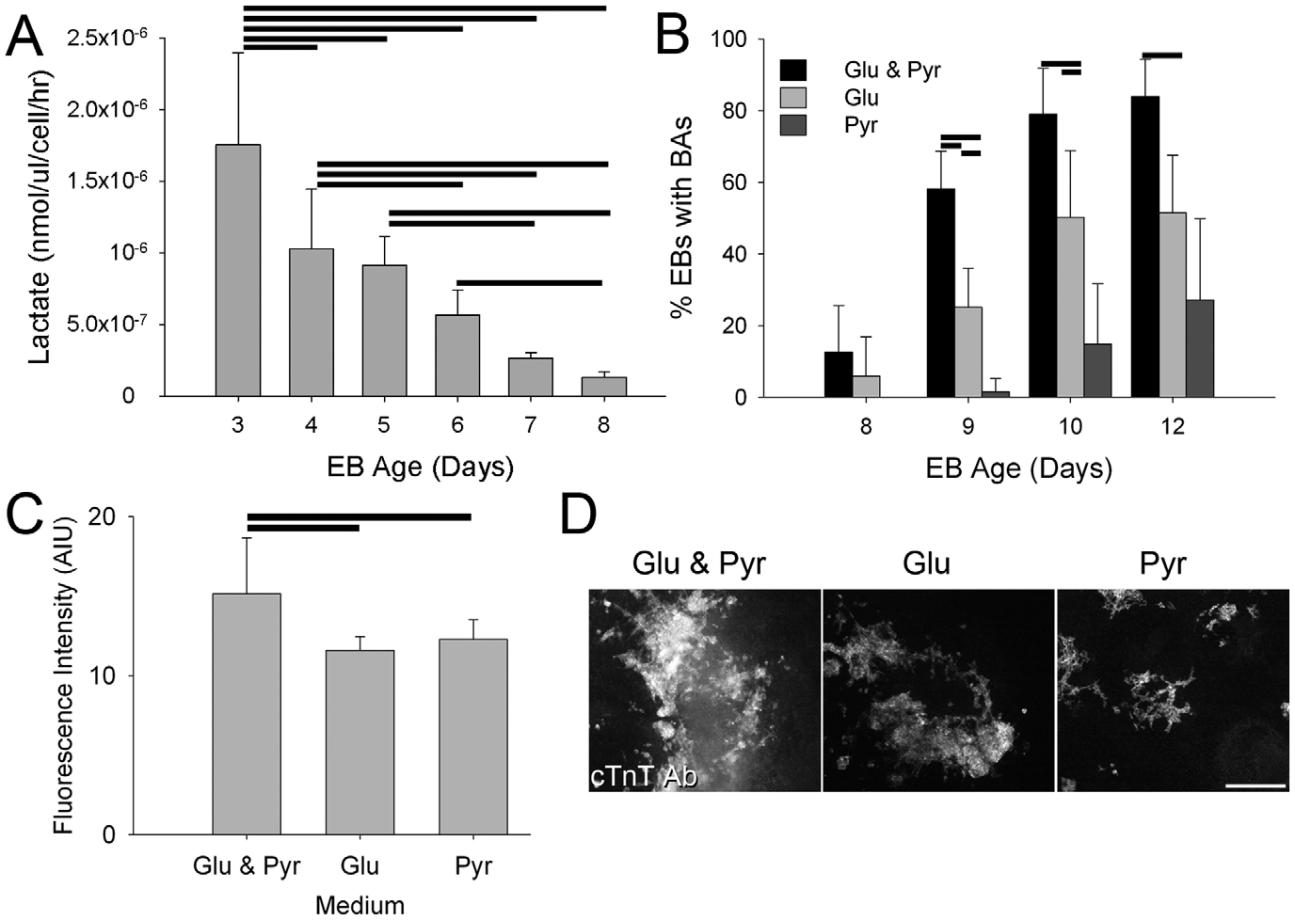
Lactate production and metabolic substrate requirements change with differentiation. A. Lactate production as detected in the medium on days 3 through 8 of mEB development. Horizontal lines indicate statistical difference (*P*<0.05) (Holm-Sidak method pairwise multiple comparison following repeated measures ANOVA). B. Percentage of mEBs that exhibited beating areas when cultured in either standard medium (Glu & Pyr), medium with glucose only (Glu) or medium with pyruvate only (Pyr) as assessed on days 8, 9, 10 and 12 after EB formation. Horizontal lines indicate statistical difference (*P*<0.05) (Tukey test following ANOVA). C. Quantitation of cTnT antibody labeling using fluorescence intensity measures from EBs grown with glucose and pyruvate, glucose only or pyruvate only. Horizontal lines indicate statistical difference (*P*<0.05) (Tukey pairwise comparison test following ANOVA). D. Examples of cTnT antibody labeling in EBs cultured in the 3 media. Scale bar = 500 µm.

To determine whether specific metabolic parameters change during the same developmental time window as observed differences in fluorescence intensity and lifetime of NADH, we examined lactate production as a measure of glycolytic flux versus mitochondrial function. We assayed the amount of lactate produced and released into the medium of mESC and mEB cultures daily from 3 to 8 days after EB formation (Fig. 2A). We found that the amount of lactate produced per cell per hour in the medium significantly decreased (*P*<0.001) from early mEBs (days 3 and 4 after mEB formation: day 3 = 1.8×10^−6^±6.4×10^−7^, day 4 = 1.0×10^−6^±4.2×10^−7^ nmol/µl/cell/hr, n = 6 wells per day) to late mEBs (day 7 and 8 after formation: day 7 = 2.7×10^−7^±4.0×10^−8^; day 8 = 1.3×10^−7^±3.8×10^−8^ nmol/µl/cell/hr; n = 6 well per day). This indicates that metabolic changes associated with the production of lactate vary over the same developmental time period during which we see changes in endogenous fluorescence. This suggests that as mEBs age and differentiate, relatively more emphasis is placed on aerobic rather than anaerobic metabolic pathways.

To assess whether forced utilization of aerobic metabolic pathways inhibits functional stem cell differentiation, we tested the impact of metabolic substrate on the formation of beating areas, as an indicator of the differentiation of functional cardiomyocytes, within the mEBs. We cultured mESCs in either glucose and pyruvate medium (DMEM with 25 mM glucose and 1 mM pyruvate), glucose only medium (DMEM with 25 mM glucose and 0 mM pyruvate) or pyruvate only medium (DMEM with 0 mM glucose and 1 mM pyruvate). After 3 days, the mESCs were used to form mEBs in hanging drops and then plated. The cells were maintained throughout in the original glucose, pyruvate or glucose with pyruvate media.

Because pyruvate enters the metabolic pathway at the end of glycolysis, cells that are unable to utilize aerobic metabolism will not thrive in the pyruvate only treatment. We found that the mESCs showed no obvious morphological differences regardless of medium, with no decrease in the number of cells present in pyruvate or glucose only media after 3 days of culture (glucose and pyruvate = 3.9×10^6^; glucose only = 5.3×10^6^; pyruvate only = 4.0×10^6^). This result, that mESCs can proliferate in the absence of glucose, suggests that mESCs are capable of utilizing aerobic metabolism, at least when forced to do so as in the pyruvate only treatment. There were also no overt differences in mEB formation or attachment in the different media, however, there was a marked reduction in the percentage of mEBs that produced beating areas in either the glucose only or the pyruvate only media (Figure 2B). At day 9 after mEB formation, a significantly higher proportion of mEBs exhibited beating areas in the glucose and pyruvate medium (0.79±0.13, n = 59 mEBs) than in either the glucose only (0.25±0.12, n = 67 mEBs; *P*<0.001) or the pyruvate only medium (0.02±0.05, n = 64 mEBs; *P*<0.001; Tukey multiple comparison test following ANOVA). Although the number of beating areas increased in all treatments through day 12 after mEB formation, the proportion of mEBs with beating areas remained significantly lower in the pyruvate only medium compared to the glucose only and glucose with pyruvate medium. Furthermore, the amount of cardiomyocytes produced by mEBs in either the glucose only or the pyruvate only media is reduced (*P*<0.001, Kruskal-Wallis ANOVA; n ≥8 EBs per treatment), as assessed by the fluorescence intensity of cells labeled with an antibody for the cardiomyocyte protein cardiac troponin T (cTnT) (Fig 2 C, D). This indicates glucose, and therefore likely glycolysis and anaerobic metabolic pathways, is necessary for the efficient production of functional cardiomyocytes, though whether this is due to a direct need of the cells for the energy produced via glycolysis or a need for other byproducts of glycolysis in unclear. Regardless of the mechanism, this result supports the hypothesis that changes in endogenous fluorescence with EB development and differentiation of cell types such as cardiomyocytes are associated with changes in preferred metabolic pathways.

### mESCs Driven to Differentiate with Retinoic Acid also Exhibited a Global Increase in Intensity but Minimal Change in Fluorescence Lifetime

Our intention was to globally assess changes in fluorescence lifetime parameters in ESC cells with the loss of pluripotency. However, these images indicate cellular and subcellular regional differences in the metabolic state of these cell aggregates (Fig. 1B). Particularly striking was the presence of single or groups of round, high intensity, cells frequently found on the edges of mEBs and increasing with mEB age, that exhibit not only extreme fluorescent lifetimes but also poor fits of the lifetime model. Although the status of these cells is unknown, we speculate that these may be dead or dying cells since dying cells can show an increase in NADH fluorescence [23], [24], [46]. There were also regions of both pluripotent colonies and mEBs that exhibited slightly longer or shorter lifetimes, though no consistent pattern in these differences could be discerned. These differences may be indicative of constitutive fluctuations in metabolism within the aggregate, or may reflect difference in cell state of individual cells.

The variability of fluorescence lifetime observed for mEB differentiation could be attributed to differences in the temporal progression of individual cells undergoing spontaneous differentiation. In an effort to reduce this variable, mESCs were treated with retinoic acid (RA) to enhance differentiation toward a neural fate [47]–[49]. Treated cells were examined after 0 (untreated control), 3 or 6 days of RA exposure. Although the intensity values were highly variable, the trend in endogenous fluorescence intensity was similar to that identified using the EB formation method of spontaneous differentiation. Specifically mESCs treated with RA showed an increase in fluorescence intensity with time of treatment compared to untreated controls, which was significant on day 6 of treatment (*P* = 0.044; day 0 = 6.1±6.2 AIU, n = 23 regions; day 6 = 8.1±6, n = 18 regions; Mann-Whitney) (Fig. S3). However, unlike the spontaneously differentiated mESCs, significant differences in fluorescence lifetime (τ_m_) were not detected with RA treatment, even at day 6 of treatment (*P = *0.86; day 0 = 1.36±0.08 ns, n = 24 regions; day 6 = 1.36±0.064 ns; n = t-test) (Fig. S3), although Oct4 levels, based on antibody staining, were significantly reduced with treatment, compared to day 0 (day 0 = 102.4×10^4^±88.5×10^4^ AIU per nucleus, n = 24 regions; *P*≤0.001; day 3 = 43.3×10^4^±30.9×10^4^ AIU per nucleus, n = 19 regions; *P* = 0.006; day 6 = 47.8×10^4^±39.7×10^4^ AIU per nucleus, n = 14 regions; Mann-Whitney; Fig. S3). With this global method it was not possible to directly correlate endogenous fluorescence parameters with levels of Oct4 antibody labeling. Because of the temporal delay and morphological changes caused by fixation, direct alignment between the specific optical section of the endogenous fluorescence images and the fixed images was not possible (Fig. S3 H, I). Thus changes detected in endogenous fluorescence lifetime and intensity detected in this experimental schema may not directly correlate with changes in Oct4 protein expression and may, instead, be due to the inability to correlate global changes in a single optical section (endogenous fluorescence in living cells) to global changes of a stack of optical sections (immunofluorescence of Oct4 in fixed cells).

### Initiation of Differentiation on a Cellular Level was Associated with Changes in Endogenous Fluorescence in Mouse ESCs

Because global changes were detectable in endogenous fluorescence intensity and lifetime in spontaneously differentiated mESCs yet such global changes could not be correlated with global changes in the pluripotency marker Oct4, we compared these endogenous fluorescence parameters with changes in a pluripotency marker at the level of individual cells. We utilized a mouse ESC line expressing an Oct4-promoter driven GFP as a readout of pluripotency [50]. With this line, immediate sequential image collection of GFP intensity using 890 nm excitation (reflecting pOct4-GFP expression) and endogenous fluorescence intensity and lifetime using 780 nm could be accomplished in living mEBs. Emission filters were also used to spectrally separate the endogenous intensity and lifetime from the GFP fluorescence (see Methods). These emission filter sets were tested on high GFP expressing Oct4-GFP mESCs to show that there was essentially no spectral overlap between fluorescent signals (Fig. S4).

**Figure 3:**
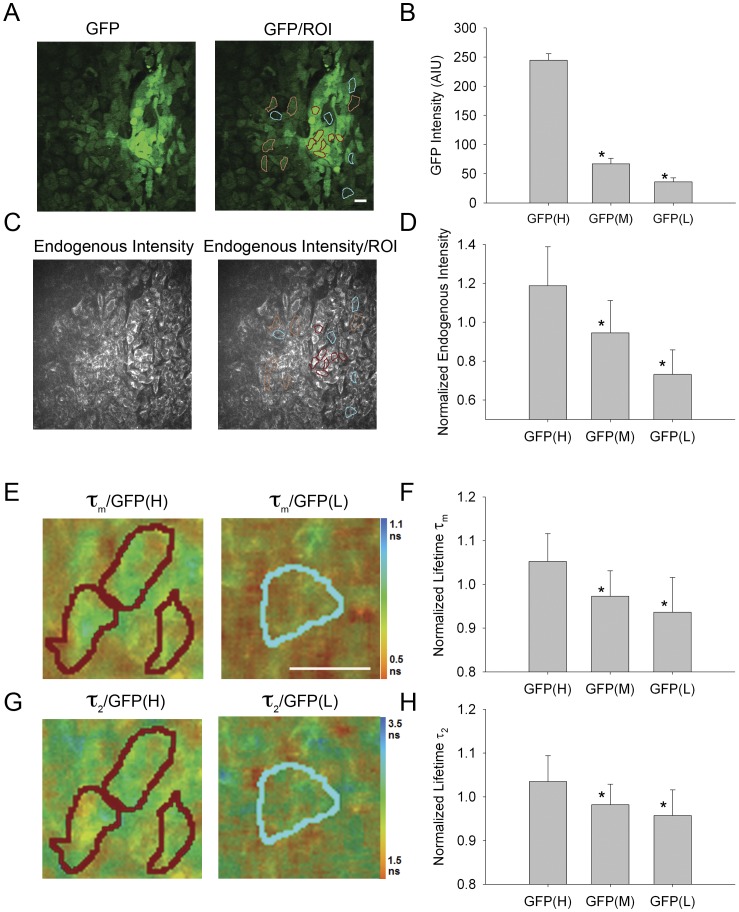
Cell level changes in endogenous fluorescence intensity and lifetime with loss of pluripotency of mESCs. A. GFP intensity of MPLSM single optical section of live mESCs expressing the pOct4:GFP transgene at 5 days post-EB formation using 890 nm excitation and 520/35 nm emission filter. Right image shows regions of interest (ROI), roughly corresponding to single cells, with high intensity GFP (GFP(H)) outlined with red; medium intensity GFP (GFP(M)) outlined with orange; low intensity GFP (GFP(L)) outlined with blue. B. Quantitative assessment of ROIs of GFP intensity. C. Intensity images of the same MPLSM single optical section as (A) using 780 nm excitation and 457/50 nm emission, corresponding primarily to NADH endogenous fluorescence. Right images show same ROIs as (A). D. Quantitative assessment of ROIs of endogenous intensity. E. Color mapped mean lifetime (τ_m_) images of endogenous fluorescence showing the same ROIs, with quantitative assessment in F. G. Color mapped long lifetime (τ_2_) images of endogenous fluorescence with quantitative assessment of ROIs (H). Scale bars = 20 µm. Color bar indicates lifetime values for τ_m_ (E) or τ_2_ (G). * indicates significant difference between GFP(H) ROIs and ROIs of medium or low GFP intensity (*P*≤0.001; ANOVA on ranks followed by Tukey multiple pairwise comparison test at *P*<0.05).

Initial time course studies indicated that the most dramatic change in GFP expression in this cell line occurred between days 4 and 6 after mEB formation ([51]; data not shown) so we concentrated our analysis on day 5 mEBs. We defined cells with high GFP expression (>200 AIU) as “GFP(H)”, those with reduced GFP expression (50–100 AIU) as “GFP(M)” and those with minimal GFP expression (<50 AIU) as “GFP(L)” (Fig. 3A, B). These three categories of regions of interest (ROIs) exhibited significantly different GFP intensity levels (*P*≤0.001; GFP(H) = 244.563±11.288 AIU, n = 32 ROIs; GFP(M) = 67.065±9.196 AIU, n = 16 ROIs; GFP(L) = 36.123±6.979 AIU; n = 20 ROIs; one way ANOVA on ranks; Dunn’s method all pairwise comparisons different at *P*<0.05). Cells with low GFP expression had lower NADH intensity than those with high GFP expression (*P*≤0.001; GFP(H) = 1.19±0.2 normalized intensity, n = 32 ROIs; GFP(L) = 0.73±0.1 normalized intensity, n = 19 ROIs; Tukey test following ANOVA) (Fig. 3C, D), indicating that cells with a diminished expression of the Oct4 pluripotency marker also exhibited a decrease in the predominantly NADH endogenous fluorescence. To further identify the relationship between the levels of Oct4-GFP and endogenous fluorescence, we determined the correlation coefficient for Oct4-GFP intensity and each endogenous fluorescence parameter. For endogenous fluorescence intensity the correlation coefficient was 0.7226.

Furthermore, this distinction between pluripotent (GFP(H)) and differentiating cells (GFP(L)) could be distinguished with fluorescence lifetime. The differentiating cells exhibited shorter τ_m_, τ_1_, and τ_2_ values compared to the pluripotent cells (Fig. 3E–H, Fig. S5) (normalized τ_m_: *P*≤0.001; GFP(H) = 1.05±0.06; GFP(L) = 0.936±0.08; normalized τ_1_: *P*≤0.001; GFP(H) = 1.04±0.07; GFP(L) = 0.96±0.05; τ_2_: *P*≤0.001; GFP(H) = 1.04±0.06; GFP(L) = 0.96±0.06 ns; Tukey test following ANOVA; GPF(H) n = 32 ROIs; GFP(L) = 19 ROIs). The correlation coefficients with GFP intensity were: τ_m_ –0.617; τ_1_–0.504; τ_2_–0.528. The lifetime parameter α_2_, corresponding to the contribution of the longer lifetime component (largely bound NADH) to the overall fluorescence decay, decreased, though not significantly, when comparing GFP(H) to GFP(L) cells (*P* = 0.072; Kruskal-Wallis ANOVA) and the correlation coefficient for α_2_ and GFP intensity was only 0.279. Interestingly, for all endogenous fluorescence lifetime parameters, cells deemed to have intermediate GFP levels exhibited values similar to those with low GFP levels but different than those with the high GFP levels (for normalized τ_m_, τ_1_, τ_2_, and α_2_: *P*≤0.05; one way ANOVA followed by Tukey’s test for GFP(H) vs. GFP(L) and GFP(H) vs. GFP(M) but not GFP(M) vs. GFP(L); GPF(H) n = 32 ROIs; GFP(M) = 17 ROIs; GFP(L) = 19 ROIs). This suggests either that the GFP was more stable than the Oct4 protein and thereby detected for a period after the loss of the Oct4 expression or the processes reflected by these endogenous fluorescence lifetime parameters may be sensitive to the initial “turn down” of Oct4 expression when the switch from pluripotency to a more determined lineage state occurs.

### Endogenous Fluorescence Intensity and Lifetime Change with hEB Differentiation at the Global and Cellular Scale

**Figure 4:**
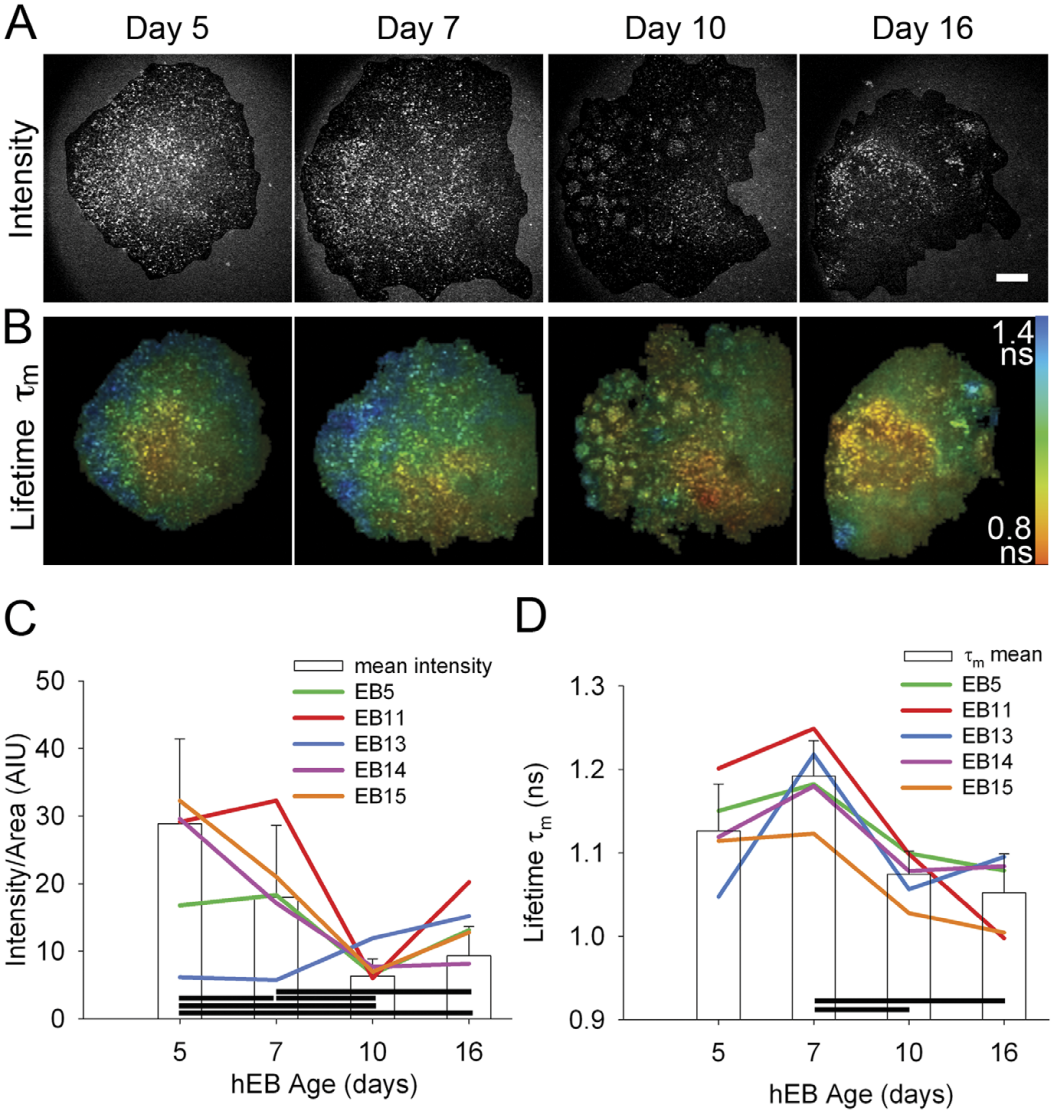
Global endogenous fluorescence intensity and lifetime changes observed with differentiation of human EBs. A. Background subtracted intensity of MPLSM single optical sections of the same live hEB at 5, 7, 10 and 16 days post-EB formation, using 780 nm excitation. B. Color mapped fluorescence lifetime (τ_m_) of the same hEB as in upper row, over time. Color bar indicates lifetime values for τ_m_. Scale bar = 50 µm. C. Quantitation shows changes in mean fluorescence intensity at the different time points (bars: for days 5, 7, 10, 16 n = 16, 17, 17, 16 EBs, respectively) while colored lines indicate the changes in intensity of individual EBs tracked over time. D. Quantitation of τ_m_, with bar graph showing the mean τ_m_ at the given time points (for days 5, 7, 10, 16 n = 5, 6, 6, 5 EBs, respectively), while colored lines show the changes in lifetime of individual hEBs tracked over time. Horizontal bars indicate differences at *P*<0.05 (Holm-Sidak method for pairwise comparisons following repeated measures ANOVA). Quantitation of additional lifetime parameters and detailed statistics in Figure S6.

Since changes could be detected in both the global and cellular scale endogenous fluorescence intensity and fluorescence lifetime with the temporal progression of mouse EB differentiation, we wished to determine whether trends would be maintained between species. We were particularly interested in studying changes with differentiation of clinically relevant, human stem cells. Because human EBs are more difficult to produce and more heterogeneous than their murine counterparts, we performed a global analysis of endogenous fluorescence intensity on individual human EBs (hEBs) starting at day 5 of EB formation. We found that, unlike in the mouse, the global fluorescence intensity decreased with hEB age from day 5 through day 10 (*P*≤0.001; day 5 = 28.8±12.6 AIU, n = 16 EBs; day 10 = 6.3±2.5 AIU, n = 17 EBs; one-way repeated measures ANOVA) (Fig. 4A, C; Fig. S6). When the intensities of a subset of individual EBs were graphed over time, some followed this trend, but some did not, indicating the existence of variability in that intensity that could be exploited by sorting technologies. This subset of hEBs was tracked over time for fluorescence lifetime following 780 nm excitation. The τ_m_ decreased over time (*P*≤0.001; day 5 = 1.13±0.056 ns, n = 5 EBs; day 16 = 1.05±0.047 ns, n = 5 EBs; one way repeated measures ANOVA) (Fig. 4 B, D; Fig. S6). This decrease in τ_m_ was similar, though delayed, compared to the trend observed with mouse EBs. τ_2_ also showed decrease over time (P≤0.001; repeated measures ANOVA; Fig. S6) while changes in τ_1_ and α_2_ were not significant (*P* = 0.170 and *P* = 0.217, respectively, one way repeated measures ANOVA; Fig. S6).

**Figure 5:**
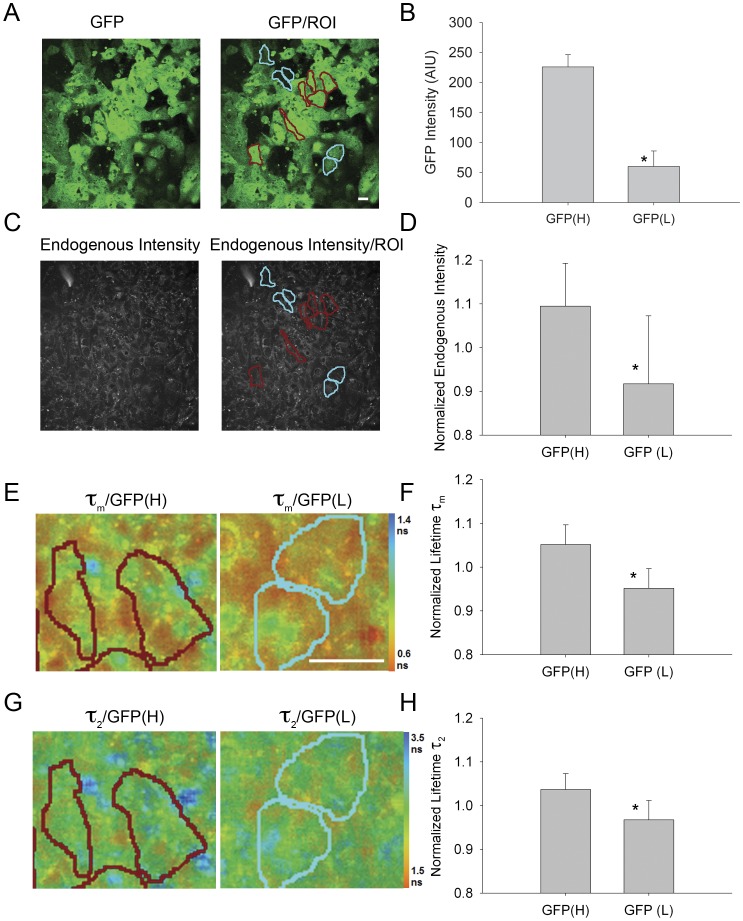
Cellular level changes in endogenous fluorescence intensity and lifetime with loss of pluripotency of hESCs. A. GFP intensity of MPLSM single optical section of live hESCs expressing pOct4:GFP: at 10 days post-EB formation using 890 nm excitation and 520/35 nm emission. Right image shows regions of interest (ROIs), roughly corresponding to single cells, with high intensity GFP (GFP(H)), outlined in red; low intensity GFP (GFP(L)), outlined in blue. B. Quantitative assessment of ROIs of GFP intensity. * indicates significant difference between GFP(H) ROIs and GFP(L) ROIs (*P*≤0.001). C. Intensity of the same MPLSM single optical section as (A) using 780 nm excitation and 457/50 nm emission corresponding primarily to NADH endogenous fluorescence. Right image shows same ROIs as (A). D. Quantitative assessment of ROIs of endogenous intensity. E. Color mapped mean lifetime (τ_m_) images of endogenous fluorescence with quantitative assessment of ROIs (F). G. Color mapped long lifetime (τ_2_) images of endogenous fluorescence with quantitative assessment of ROIs (H). Scale bars = 20 µm. Color bar indicates lifetime values for τ_m_ (E) or τ_2_ (G). * indicates significant difference at P<0.01 (t-test).

To monitor cellular scale trends, we utilized an Oct4-promoter driven GFP human ESC line. With this cell line, immediate sequential image collection of GFP intensity (reflecting Oct4-GFP expression) and endogenous fluorescence intensity and lifetime could be accomplished in living hEBs using multiphoton microscopy at 890 nm excitation for GFP expression and 780 nm for endogenous fluorescence intensity and lifetime (see Methods). The time window of development showing the most intra-aggregate variability in GFP expression was found to be day 10 after hEB formation, and therefore, this day was chosen as the time point for cellular analysis (Fig. 5). We defined cells with high GFP expression (as a reflection of high Oct4 expression) (>200 AIU) as “GFP(H)”, those with minimal GFP expression (<100 AIU) as “GFP(L)” (Fig. 5A, B). We found that cells with low GFP expression (differentiating) had lower NADH intensity than those with high GFP expression (*P* = 0.004; GFP(H) = 1.1±0.1 normalized intensity, n = 11 ROIs; GFP(L) = 0.92±0.2, n = 11 ROIs; t-test) (Fig. 5C, D) and the correlation coefficient was 0.596. Furthermore, declines in τ_m_, τ_1_ and τ_2_ were observed when comparing the GFP(L) cells to the GFP(H) cells (normalized τ_m_: *P*≤0.001; GFP(H) = 1.05±0.05, GFP(L) = 0.95±0.05; normalized τ_1_: *P* = 0.002, GFP(H) = 1.04±0.04, GFP(L) = 0.96±0.05; normalized τ_2_: *P* = 0.001, GFP(H) = 1.04±0.04, GFP(L) = 0.97±0.04 ns; GFP(H) n = 11 ROIs; GFP(L) = 11 ROIs; t-test or Mann-Whitney) (Fig. 5E–H, Fig. S7). The correlation coefficients for these parameters with GFP intensity were: τ_m_ –0.778, τ1–0.719, τ_2_–0.695. This analogous trend of decreasing endogenous lifetimes during an early phase in the loss of pluripotency found in both the mouse and human ES cell lines suggests a metabolic change that, with further exploration, may provide insight into the metabolic mechanism(s) associated with the loss of pluripotency.

### Potential Utility of Endogenous Fluorescence Parameters for Identifying Cell State

**Figure 6:**
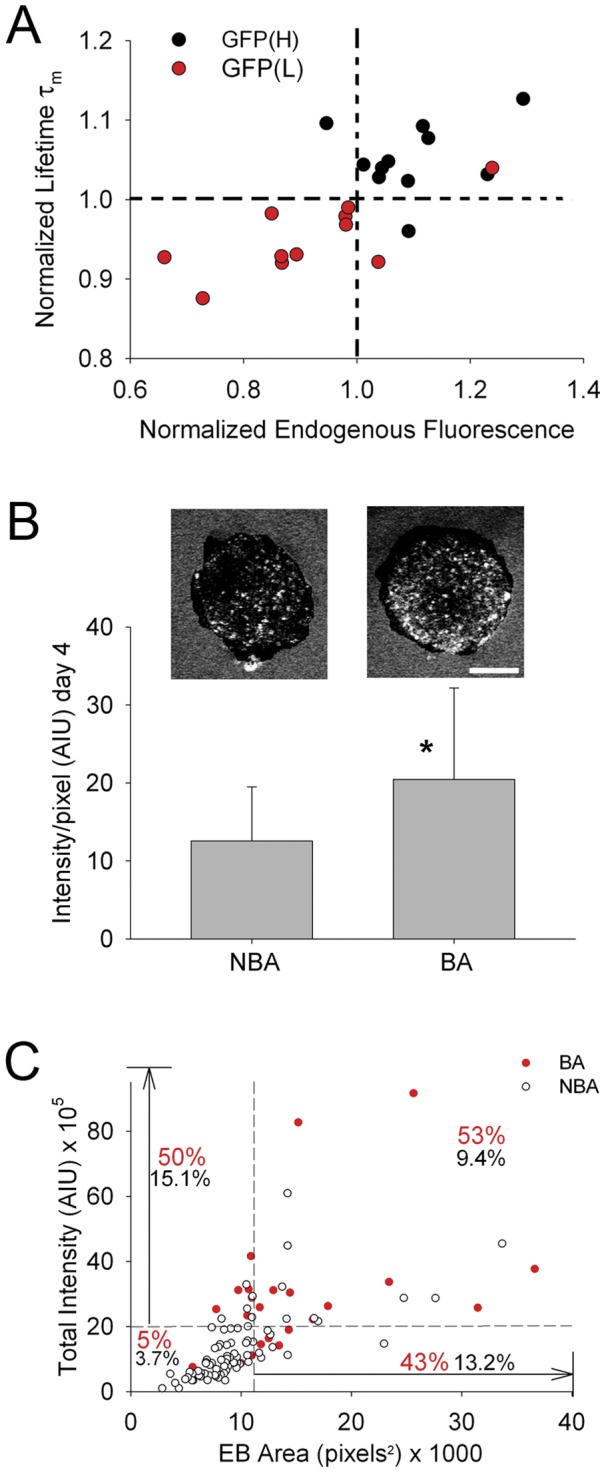
Potential utilization of endogenous fluorescence parameters. A. Scatter plot showing relationship between fluorescence lifetime (τ_m_), endogenous fluorescence, and Oct4-GFP intensity category. Each circle represents one cell. Dotted lines indicate arbitrary threshold to illustrate the effect of segregating a population of cells based on two endogenous fluorescence parameters. B. Quantitation comparing the endogenous fluorescence intensity (background subtracted intensity per pixel of EB brightfield area) of hEBs on day 4 of EB formation, segregated by those that subsequently formed beating areas (BA) and those which did not (NBA). * indicates significant difference (*P*≤0.001, Mann-Whitney). Images above bars show MPLSM single optical sections of hEBs, using 780 nm excitation, on day 4 after hEB formation prior to attachment, to show background subtracted endogenous fluorescence intensity. hEB on right subsequently develops a beating area (BA) while the one on the left does not (NBA). Scale bar = 100 µm. C. Assessment of the putative enrichment for subsequent developmental of beating areas (day 24) based on fluorescence intensity on day 4. Graph shows the proportion of hEBs that developed beating area (filled red circles) when selected from the 30% of EBs that have the highest fluorescence intensity at day 4 (above horizontal dotted line), largest area (to right of vertical dotted line) or both (upper right quadrant) compared to those in the lower 70% (below or to left of dotted lines for intensity or area, respectively). Open circles represent EBs that did not develop beating areas. Red numbers indicate percentage of EBs with beating areas in that category (*e.g.* upper 30% of intensity or upper 30% of area) of the total number of EBs *within that category* while black numbers indicate the percentage of EBs with beating areas in that category of the *total* number of EBs examined (n = 106 EBs).

Given the strong correlations observed between endogenous fluorescence parameters and changes in the levels of the pluripotency marker Oct4, we were interested in whether a combination of two parameters might be useful for segregating pluripotent cells, as a prelude to considering these endogenous fluorescence parameters as tools for identifying cells of differing fates. When endogenous intensity and τ_m_, which have correlation coefficients with GFP intensity of 0.596 and 0.778, respectively, are graphed relative to one another (Fig. 6A), the cells with the high levels of GFP intensity cluster in the upper right quadrant. This way of visualizing the data illustrates the utility of how two endogenous fluorescence parameters could be used in conjunction to improve the robustness of endogenous fluorescence as a marker for cell status.

To extend this concept, we sought to assess whether endogenous fluorescence could be used as a non-invasive method to identify hEBs with particular potential, we determined whether fluorescence intensity at an early developmental time point would correlate with the subsequent formation of beating areas, indicative of the formation of contraction-competent cardiomyocytes. To test this possibility, on the first day of plating (day 4 of hEB formation), prior to attachment, the endogenous fluorescence, with 780 nm excitation, of hEBs in individual wells was collected. These individual hEBs were assessed at day 24 after EB formation for the presence of beating areas (foci of spontaneously contracting cells). When the intensity is normalized to EB size (as determined from brightfield images), hEBs that develop beating areas had significantly higher endogenous fluorescence intensity per area, than those which did not (*P*≤0.001, Mann-Whitney; with beating areas = 20.45±11.7 AIU/pixel, n = 24 EBs; without beating areas = 12.7±7.1 AIU/pixel; n = 82 EBs) (Fig. 6B) indicating that increased endogenous intensity, on day 4, correlated with the subsequent development of beating areas.

Because EB size can influence hEB development and beating area formation [52] we compared the overall intensity, above background, of day 4 hEBs that developed beating areas to those that did not. The total intensity was still significantly higher in EBs that formed beating areas than those that did not (p<0.001; beating areas = 28.7×10^5^±20.2×10^5^ AIU, n = 24; no beating areas = 13.1×10^5^±10.5×10^5^ AIU; n = 82). To assess the possibility of using higher endogenous fluorescence as a parameter for enriching a population of hEBs for beating area, these hEBs were organized by total fluorescence intensity. The population of hEBs with the top 30% of total intensity values was enriched for hEBs with beating areas (50% have beating areas, n = 32 EBs) compared to the proportion of hEBs with beating areas in either the entire population (*P* = 0.006; 22.6% have beating areas, n = 106 EB; Chi-square) or the population in the lower 70% (*P*≤0.001; 10.8% have beating areas, n = 74 EBs; Chi-square). This enrichment indicates that sorting hEBs at day 4 by endogenous intensity could be used to enrich for hEBs that will produce cardiomyocytes (Fig. 6C). If the hEB data were “sorted” by both endogenous fluorescence intensity and EB size the percentage of hEBs with beating areas in this “sorted” population (upper right quadrant) was increased slightly (*P* = 0.91; 52.6% have beating areas, n = 19 EBs; Chi-square).

## Discussion

Here we employed a multimodal approach to discretely define parameters of endogenous fluorescence of NADH on varying spatial (*i.e*., global and cellular) and temporal (*i.e*., weeks, days and at discrete time points corresponding to loss of potency) scales characteristic of stem cells and associated progeny. We identified reproducible changes in both fluorescence intensity and fluorescence lifetime in the course of stem cell differentiation lending credence to the development of fluorescence as an endogenous, and thus noninvasive, signature capable of distinguishing pluripotent cells from more mature cells. These efforts will improve basic studies of stem cell behavior and should enable more rapid, regulatory clearance of stem cells for clinical use.

We found that fluorescence *intensity* of NADH of regions of mESC-derived EBs increased as a function of time under conditions conducive to differentiation. Interestingly, the trend is inverted if one confines the analysis to individual cells of mESC-derived EBs, at stages corresponding to loss of pluripotency (*i.e*., diminished Oct4 expression). This discrepancy has several possible explanations. First, global analysis of regions of EBs may be confounded by the heterogeneity of cell states of the populations studied. For example, dying cells are known to harbor high levels of NADH [23], [24], [46]; this increasing NADH tends to accumulate in mitochondria in the perinuclear space and is thought to enable nuclear involution with apoptosis [23], [24]. A variety of studies [53]–[55] and our own observations suggest that EB undergo cell death as they differentiate and so the observed global increase in NADH with differentiation may correspond to the accumulation of dying cells and not to differentiation *per se*. In contrast, the cell-by-cell analysis specifically selected morphologically viable cells. Alternatively, the heterogeneity of the cells types produced may alter the NADH intensity levels observed. For example, the mESC line and corresponding differentiation medium used in this study are known to generate a high proportion of cardiomyocytes, which contain a high density of mitochondria relative to other mature cell types [56]. Thus the observed increase could correspond largely to the generation of particular mature cell types and may not be apparent in the early phases of differentiation corresponding to the loss of Oct4 expression studied in the cell-by-cell analysis. It is quite possible that the long-term trend of endogenous fluorescence in mEBs does not reflect the short-term individual cellular trend during the pluripotency transition.

Do these caveats negate the possibility of using changes in NADH intensity to delineate changes in potency? We suggest NADH intensity is still a valuable parameter, but should be accompanied by proper controls to eliminate the impact of cell death and should ideally be coupled with other components of the fluorescent signal (*i.e*. fluorescence lifetime and possibly spectral imaging [57]) to help validate and confirm the signal and the changes observed. In addition, potential differences between species, between developmental time points, between differentiation protocols as well as between stem cell isolates of varying potency should be taken into consideration. For example, in this study we find that global endogenous fluorescence intensity trends differ between mouse and human EB differentiation while recent reports show NADH intensity increases with differentiation of hMSCs [37], [38], [58] and decreases with differentiation of human salivary gland stem cells [40]. Recent work has shown that fluorescence lifetime can be used to discriminate between pluripotent and differentiating human stem cells and found NADH concentration increased with differentiation [41] while our examination of NADH intensity suggests that NADH levels decrease with hEB age. However, the two studies are not directly comparable given several differences in methodology: 1. the approaches for generating the fluorescent lifetime data (phasor vs time correlated single-photon counting); 2. the time window of development was different (0 to 4 days of differentiation vs 5 to 16 days); 3. the differentiation culture (monolayer forced differentiation vs EB spontaneous differentiation). Even in studies of cell death there are differences in the endogenous fluorescence response [59], likely due to the array of factors that contribute both to the acquisition of the endogenous fluorescence as well to variation in the biology of cell death. For example, the temporal window of examination has a significant impact on the information derived from the fluorescence. In the early phase of apoptosis, there is a transient increase in fluorescence intensity that subsequently dramatically decreases [23]. Furthermore, whether the metabolic insult triggering cell death processes is recoverable or not can also impact the endogenous fluorescence [24]. This variability does not reduce the value of the information gleaned from the endogenous fluorescence but rather indicates that standards may vary amongst cellular situations and it is important to recognize and identify these differences.

**Figure 7:**
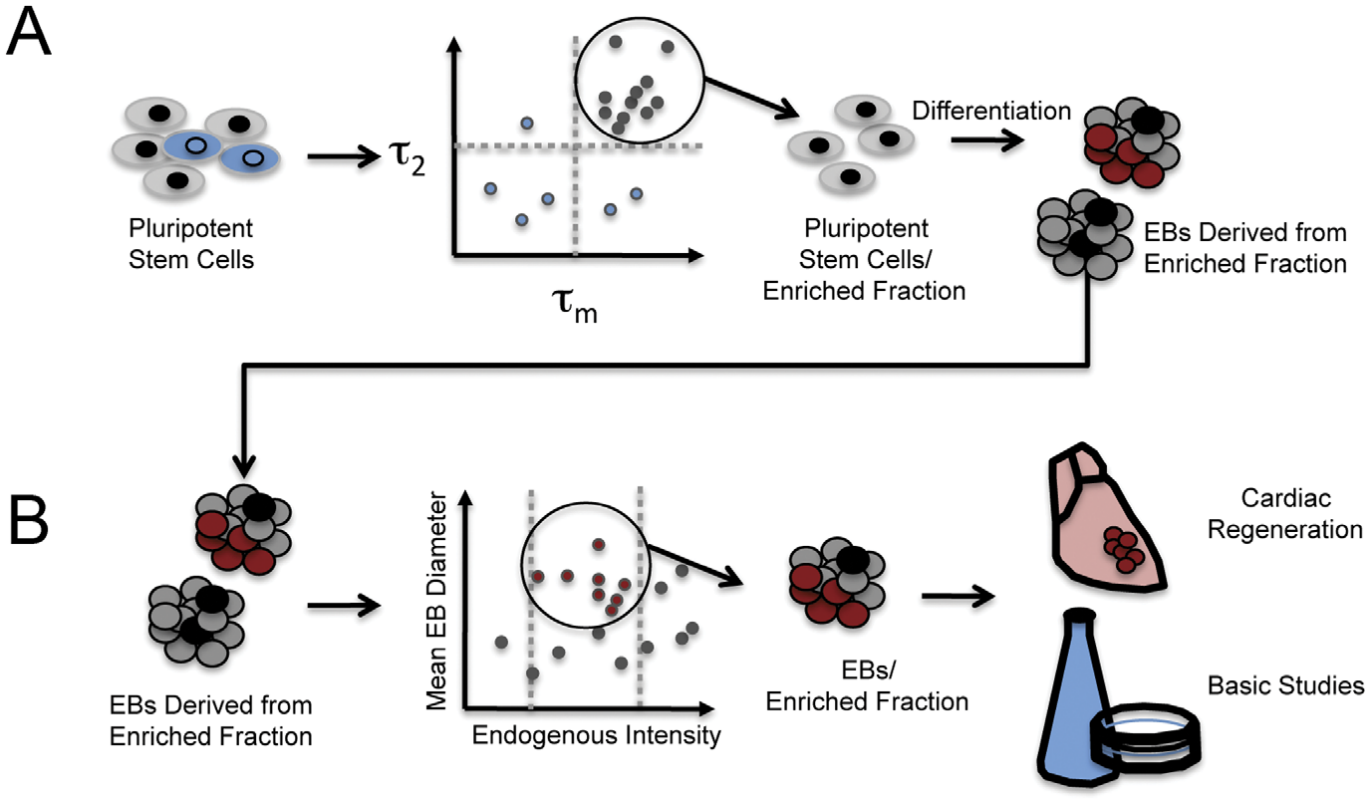
Defining signatures for non-invasive enrichment of pluripotent stem cells and associated progeny. This schematic outlines how the changes in endogenous fluorescence that occur with stem cell differentiation might be practically employed to enable both basic studies of the cues that dictate stem cell differentiation as well as clinical applications. A. Enrichment of pluripotent stem cell populations. It is difficult to maintain a pluripotent stem cell population in a culture dish as multiple stimuli can conspire to induce unwanted differentiation. Several approaches have been employed in an attempt to monitor and maintain pluripotent populations, but all are invasive in nature (i.e., exogenous labels or stably integrated antibiotic resistance genes). Instead, using a multiphoton-based flow cytometry system [5], threshold values (dashed lines) for intrinsic fluorescence lifetime elements might be identified and utilized to enrich populations. The cellular-scale analyses described here are particularly well suited to define threshold values. Stem cells enriched in this way could be used for a variety of applications, including the generation of EBs. B. Enrichment of EBs. EBs have long served as a developmentally-relevant format for inducing stem cell differentiation. The challenge is isolating EBs that give rise to particular differentiated cell types in an enhanced-throughput, noninvasive manner. Here we suggest use of parameters from our global-scale analyses, such as endogenous fluorescence intensity, to define thresholds that might be used to purify EBs at early time points that have a high probability of generating differentiated cell types of interest, such as cardiomyocytes. Differentiated cells enriched in this way would be quite valuable for basic studies of the cues that drive differentiation and perhaps, in the future, for regenerative therapies.

The other component of NADH fluorescence studied here, fluorescence lifetime, changed consistently with spontaneous differentiation, regardless of spatial or temporal analysis approach. The most prominent change was a decrease in the long lifetime component, which corresponds to protein-bound NADH [8]. NADH serves as an electron donor during energy production [60], serving as an electron carrier in oxidoreductase pathways [61]. In addition to its role in metabolism, the oxidized form, NAD+, can serve as a precursor to signaling molecules through a diversity of ADP-ribosyl transfer reactions. Furthermore, the balance of NAD+/NADH, or redox state, can affect cellular processes such as metabolism, signaling and transcription as well as providing a countermeasure to protect the cell from free radical damage [61]. Therefore, the changes we observe in the lifetime of the bound NADH may reflect changes in protein binding associated with the switch between metabolic states from pluripotent to a differentiated state [25], [27], [28], [31]. Alternatively, or in addition, there are proteins that bind NADH (e.g., CtBP) [62]–[64] that are known to induce changes in transcriptional regulation associated with differentiation, development and transformation pathways. Thus it will be useful in future studies to discern the intracellular location of altered lifetime signal (*i.e*., cytoplasm, nucleus or mitochondria). This decrease in fluorescence lifetime was undetectable with forced differentiation, suggesting alterations in cell metabolism due to differences in cell-cell contacts, since cells differentiated via the EB method had significant three-dimensional structure, whereas those directed to differentiate were grown in a monolayer. Therefore, we suggest that, combined, endogenous fluorescence intensity and lifetime provide a “signature” indicative of stem cell state wherein intensity might serve as a more cursory examination, while NADH lifetime may serve for subsequent finer discrimination (Fig. 7). Additionally, the endogenous fluorescence parameters of intensity and lifetime could be combined with other endogenous signals, such as second harmonic generation, which has been used to identify pluripotent cell-derived cardiomyocytes [65], for further refinement and selection.

The main goal of this study was to identify and characterize changes in endogenous fluorescence of stem cells as they differentiate and to support the biological validity of these observations, we incorporated independent assessments of metabolic function. A variety of studies have identified differences in metabolic parameters between pluripotent cells and differentiating cells [25]–[29], however we sought to assess such changes specifically over the developmental time period during which ESCs initiate differentiation and develop toward a cardiac fate. A full metabolic analysis of this time window is a study unto itself, thus we focused on characterizing a readily accessible metabolic parameter, lactate production, as a reflection of non-oxidative metabolism of pyruvate in mouse EBs over time. Consistent with work in iPS cells, which showed that differentiated cells exhibited lower lactate production in pluripotent cells compared to differentiated cells [30], we found that this metabolic indicator decreased with mEB age. This decrease in lactate production is inverse of that observed with endogenous fluorescence intensity. This relationship does not necessarily indicate a direct mechanistic link between lactate production and endogenous fluorescence intensity, since the endogenous fluorescence intensity is likely a reflection of complex changes in multiple metabolic pathways. Indeed, the endogenous fluorescence lifetime, which also changes over this time window, but with different kinetics than either the intensity or the lactate production, suggesting that the endogenous fluorescence parameters are the result of a combination of metabolic inputs. Future studies are needed to better understand the details of the metabolic mechanisms that lead to the observed changes in endogenous fluorescence parameters. However, this work and recent work from others [36], [41], [65] demonstrate the great potential of endogenous fluorescence as a means to assess cell state.

The decrease in the per cell lactate production as mEBs age suggests that the early EBs may be more heavily utilizing glycolysis and anaerobic metabolism, resulting in the production of lactate, while the older mEBs may be switching to a more aerobic metabolism such that the products of glycolysis can be moved through oxidative respiration and are, therefore, unavailable to generate lactate. Interestingly, when the major metabolic substrate is limited to pyruvate, which can only be metabolized aerobically, we find that pluripotent cells appear to behave normally but that differentiation does not proceed efficiently, at least in the context of functional cardiomyocyte differentiation. That pluripotent cells are not apparently affected by the lack of glucose seems contradictory both to our lactate production data and to the common view that pluripotent cells are generally metabolically “immature” and rely on anaerobic metabolism. However, this experiment merely shows that they are capable of aerobic respiration, but does not indicate a preference for metabolic pathway when given a choice of substrate. In fact, recent work suggests that in pluripotent human stem cells the majority of ATP is supplied by oxidative phosphorylation [31]. That cardiac differentiation does not proceed efficiently with pyruvate suggests a requirement for glucose, providing support for the intriguing idea that cell fate decisions can be influenced by altering metabolic substrates [66]. Of note, medium containing both substrates showed a marked increase in cardiac differentiation compared to either glucose or pyruvate alone. It is possible that EBs prefer glucose whereas differentiating cells have a requirement for pyruvate. Thus adding both carbon sources facilitates the entire process of differentiation better than either glucose or pyruvate alone.

Although the details of the mechanism(s) responsible for the fingerprint remain to be fully characterized, the practical utility of components of endogenous fluorescence as noninvasive markers for analysis and purification of stem cells (Fig. 7) is clear. Multiphoton-based technologies exist that might immediately utilize the information generated in this report [67], [68]. Unfortunately, single photon-based technologies would be less useful as the excitation wavelength needed would be cytotoxic. As one example, we have developed an enhanced-throughput multiphoton flow cytometry (MPFC) system that is capable of deep optical penetration of large aggregates, including EBs, in the context of flow [5]. Using this type of high-throughput technology one can imagine enriching, in a noninvasive manner, pluripotent cells or differentiating cells while still in the three-dimensional context of the EB. The non-invasive component will be especially useful for clinical applications such that the toxicity or potential side effects of a label, dye or genetic modification of cells can be avoided. In further support of the utility of this function, we show preliminary evidence that endogenous fluorescence of early phase differentiating cells may be indicative of the propensity to subsequently produce certain cell types. Indeed, higher total fluorescence intensity of early EBs positively correlated with the subsequent development of beating areas (i.e., presence of contractile cardiomyocytes). Thus, when properly controlled, endogenous fluorescence components of NADH, as detected with multiphoton-based technologies, can accurately describe stem cell state both in single cells and cell aggregates or larger three-dimensional microtissues, including engineered tissues.

## Methods

### Cell Culture

Mouse embryonic stem cell culture and embryoid body formation: Mouse ESC line D3 [69] and a pOct4:GFP-expressing mESC cell line [50] were maintained as pluripotent in DMEM (Invitrogen, Carlsbad, CA) or DMEM-Glutamax (Invitrogen) with 10% fetal bovine serum (Invitrogen), 1X MEM non-essential amino acids (Invitrogen), 6 µl/100 ml β-mercapto-ethanol (Sigma, St. Louis, MO), and penicillin/streptomycin (50 U penicillin and 50 µg/ml streptomycin) (Invitrogen). L-glutamine (2 mM) (Invitrogen) was added to DMEM-based medium. Cells were grown without a feeder layer but supplemented with 2000 U/ml Leukemia Inhibitory Factor (LIF, Chemicon-Millipore, Billerica, MA) and 10 ng/ml Bone Morphogenic Protein-4 (BMP-4, R&D Systems, Minneapolis, MN) [70]. Every three or four days cultures were dissociated using 0.05% trypsin-EDTA (Thermo Scientific, Waltham, MA) and re-plated at 2.5×10^4^ cells/ml onto 60 mm tissue culture dishes (BD Falcon, Bedford, MA) coated with 0.1% gelatin (Sigma). Embryoid bodies (mEBs) were generated using the hanging drop method [44]. Briefly, following trypsinization cells were resuspended at 1.6×10^4^ cells per ml in culture medium without growth factors and deposited in 30 µl drops onto the lid of a 100 mm petri dish and suspended over 20 ml sterile PBS (137 mM NaCl; 2.7 mM KCl, 4.3 mM NaPO4, 1.47 mM KPO4, pH 7.4; Fischer Scientific). For harvesting, hanging drops were collected with a cut 1000 µl pipette tip and plated in culture medium without growth factors onto gelatinized plates. All cell cultures were maintained at 37°C in 5% CO_2_.

Human embryonic stem cell EB formation: The approved human ESC line (H9– WA09) and the pOct4GFP/Zeocin-resistance expressing hESC line (H9 hOct4-pGZ) (WISC Bank, WiCell, Madison, WI) were cultured based on protocol SOP-SH-002 (WISC Bank) in TeSR medium (Stemcell Technologies, Vancouver, Canada). Oct4GPF/Zeo expressing ESC cells were maintained in the presence of 1 µg/ml zeocin (Invitrogen) on Matrigel (BD Biosciences, Bedford MA) coated 6-well plates. hEBs were generated from hESC single cell suspension, in the presence of 5–10 µM ROCK inhibitor Y-27632 (Calbiochem-EMD4 Biosciences, Gibbstown, NJ), using the Aggrewell™ 400 (Stemcell Technologies) method [71]. With 7.5×10^5^ cells per large well, this method generates EBs comprised of approximately 500 cells. hEBs were cultured in TeSR medium (low bFGF, 40 ng/ml) +15% FBS overnight. The next day, EBs were harvested from the Aggrewell™ 400 plate and kept in suspension in ultra-low attachment flasks or 6 well plates (Corning, Lowell, MA) in TeSR medium (low bFGF, 40 ng/ml) +15% FBS. To promote cardiomyocyte production (WiCell WISC Bank protocol SOP-CH-203C), the EBs were transferred on day 4 after EB formation to DMEM-F12 (Invitrogen) +20% FBS (Invitrogen), at which point they were plated for attachment and further differentiation. At about day 16, serum concentration was reduced to 2%. Human ES cell work was performed in accordance with the UW-SCRO guidelines.

### Lactate Assay

mESC and mEBs were cultured in standard medium in 6 well plates (BD Falcon, San Jose, CA). mESCs were plated at 5×10^4^ cells per well. For mEBs, the number of EBs per well was counted one day after harvesting, when EBs had attached, with 8 to 12 EBs per well. 600 µl samples of medium were collected from each well each day, at approximately 24 hour intervals. The samples were chilled on ice, centrifuged at 4°C for 15 min to precipitate any cell debris, 500 µl of supernatant was placed into a fresh, chilled tubes and placed at −80°C until all samples were collected. The assay was conducted using a fluorometric lactate assay kit (ab65330) (Abcam, Cambridge MA), following the manufacturer’s protocol. Fluorescence was measured using Fluoroskan Ascent FL fluorometer with 527 nm excitation/590 nm emission filter set and analyzed with Ascent Software (Thermo Electron Corporation, Vantaa, Finland). Lactate concentration (nmol/µL) was calculated based on fluorescence intensity using a standard curve processed in the same 96 well plate as the samples. Cell numbers were determined using the known number of cells counted at EB formation and assuming a doubling of cell number every 24 hrs. For each experiment, there were 3 replicate wells per time point and 2 experiments conducted. All time points for a given experiment were assayed in the same 96 well plate.

### Microscopy

Multiphoton laser scanning microscopy: MPLSM was conducted at the Laboratory for Optical and Computational Instrumentation at the University of Wisconsin-Madison. Endogenous fluorescence data for global analysis mouse time course, day 2 and day 4 EB (and corresponding ESCs) were collected on a spectral-lifetime imaging system [33], [57]. In short, the system is built around an inverted microscope (TE 2000, Nikon, Melville, NY) using illumination from a Ti:Sapphire mode-locking laser (tuning range of ∼700–1000 nm, Coherent Mira, Coherent, Santa Clara, CA) pumped by an 8 W solid-state laser (Coherent Verdi) to generate pulse widths of approximately 120 fs at a repetition rate of 76 MHz. The system has multiple detectors including a 16 channel combined spectral lifetime detector (utilizes a Hamamatsu PML-16 PMT) with a spectral detection range of 350 to 720 nm, and a H7422P GaAsP photon counting PMT (Hamamatsu) for intensity and lifetime imaging. Fluorescence lifetimes were acquired with an electronic hardware system for recording fast light signals by time correlated single photon counting (SPC-830, Becker & Hickl). All other data were collected on an multiphoton optical workstation [34], also at LOCI. This custom-built system has been previously described and, briefly, consists of an inverted microscope (Nikon TE2000, Melville, NY) with Cambridge galvos (Cambridge Technology, Billerica, MA), a MaiTai (Spectra Physics, Palo Alto, CA) Ti:Sapphire laser, a Hamamatsu H7422 GaAsP Photomultiplier detector (Hamamatsu Photonics, Bridgewater, NJ) for fluorescence intensity and fluorescence lifetime detection, and a sensitive silicon photodiode detector (Bio-Rad, Hercules, CA) for simultaneous transmission image collection. Lenses used were a Plan APO VC 20X (N.A. 0.75; Nikon Instruments, Tokyo, Japan), a CFI Apo Lambda S LWD 40X (N.A. 1.15, WD 0.61 mm; Nikon Instruments) or an S Fluor 40X (N.A. 1.3, WD 0.22 mm; Nikon Instruments). For collection of endogenous fluorescence alone (global analyses, non-GFP expressing cells), an excitation wavelength of 780 nm was used and all emitted photons were collected. For cellular analysis of GFP expressing cells, endogenous fluorescence was excited at 780 nm and emission collected through a 457/50 filter (Chroma, Bellows Falls, VT) while GFP fluorescence was excited using 890 nm and emission collected using a 520/35 nm filter (Chroma). The pixel frame size for the multiphoton intensity images was 512×512 while the fluorescence lifetime imaging microscope (FLIM) images were 512×512, 256×256 or 128×128. Acquisition was performed with WiscScan (LOCI, www.loci.wisc.edu), a LOCI developed software acquisition package that can control both the MPLSM intensity and lifetime image collection. For fluorescence lifetime measurements, photons were collected for 60 or 90 seconds for each lifetime image. For live imaging, stage temperature was maintained at 35.5°C and a minichamber (Warner Instruments, Hamden CT for 35 mm dishes, in-house built for 9×2 well microslides) on the stage was gassed with humidified 5% CO_2_ in air. For detection of fluorescently conjugated secondary antibodies, fixed specimens were imaged on the MPLSM with 890 nm excitation and either a 580 nm long pass or 520/35 nm bandpass filter (Chroma).

Fluorescence microscopy: DAPI labeled nuclei were detected using an IX71 inverted deconvolution fluorescence microscope (Olympus, Center Valley, PA). Images were acquired with 20X UPlanFluor objective (NA = 0.5) with Slidebook software (Intelligent Imaging Innovations Denver, CO). Nuclei were counted using FIJI ImageJ (http://fiji.sc).

### Experimental Design

Mouse global EB time line: To generate a time line of changes in endogenous fluorescence, mEBs were formed (day 0). The mEBs were then plated for onto gelatin-coated imaging dishes, either 35 mm glass-bottomed (World Precision Instruments, Inc., Sarasota, FL) or 35 mm optical-quality plastic (ibidi, Munich, Germany) either on day 1 (for analysis on day 1, 2 or day 4) or on day 2 (for analysis on day 3, 5, or 8). mESC were plated on imaging dishes at least 24 hrs prior to imaging on the same day as each of the mEB time points. Each dish was imaged for intensity and lifetime only once.

Mouse global forced differentiation: Mouse mESCs were cultured on 35 mm gridded imaging dishes (ibidi) for 1 day, with growth factors. One sample was imaged intensity and lifetime as the “no treatment” control (day 0), while the other samples were treated with 10^−6^ M retinoic acid (RA) (Sigma) in culture medium without growth factors [49]. Intensity and lifetime images were collected after 3 and 6 days of RA treatment, with RA being maintained during that time. Each sample was imaged only once and then fixed in 4% paraformaldehyde (EMS, Hatfield, PA) in PBS for 15 min. Cells were subsequently immunolabeled for Oct4 expression. Following blocking with 5% bovine serum albumin (BSA) (Fischer Scientific), 0.1% TritonX-100 (Fischer Scientific); 2% normal goat serum (Chemicon/Millipore), cells were incubated overnight at 4°C with 1∶200 anti-Oct3/4 antibody (Santa Cruz Biotechnology, Santa Cruz, CA). After rinsing with PBS, cells were incubated for 2 hours at room temperature with 1∶500 goat anti-mouse secondary antibody (Alexafluor 488 or Alexafluor 568, Molecular Probes/Invitrogen). After rinsing in PBS, cells in the same grid locations as those for which endogenous fluorescence images were collected were re-imaged as z-stacks on the MPLSM using appropriate filters. Cells were subsequently mounted in diazabicyclo(2,2,2)octane (DABCO) (Sigma) (2.5 mg/ml) with 4′,6-diamidino-2-phenylindole (DAPI) (50 µg/ml) in 50% glycerol (Fischer Scientific) and 50% PBS. The same grid locations were imaged for DAPI on the deconvolution fluorescence microscope (IX71 Olympus) to determine number of nuclei.

Human global EB time course: On day 4 after hEB formation, hEBs were plated individually into the small wells of 2×9 optical-quality plastic microslide (ibidi) in approximately 70 µl of culture medium. Each hEB was imaged for endogenous fluorescence intensity while in suspension in the wells. hEBs were returned to the incubator and allowed to attach overnight. The next day, additional medium was added, to approximately 800µl in each of the 2 large wells, so that medium was contiguous between 9 of the small wells. A subset of these EBs was consecutively imaged for endogenous fluorescence intensity and lifetime on day 5, day 7, day 10 and day 16. Cultures were maintained and the presence of beating areas was assessed at day 24.

Mouse and human cell by cell analysis: Mouse or human EBs were individually plated in 2×9 optical-quality plastic microslides or as small groups in 35 mm imaging dishes (ibidi), respectively, and imaged on day 5 after mEB formation and day 10 after hEB formation. GFP fluorescence images, endogenous fluorescence intensity and endogenous lifetime images were collected as single optical sections of the same field of view for each EB, 15 µm from the bottom of the mouse EBs and from the central section of the human EBs. Each EB was imaged first for GFP fluorescence at 890 nm with a GFP filter (520/35 nm) (Chroma) and then immediately switched to 780 nm with an endogenous filter for NADH (457/50 nm) [16] (Chroma) to collect endogenous intensity and lifetime. The fluorescence lifetime instrument response was measured for each objective and filter combination utilized in the imaging by collecting the lifetime response of solid urea crystals for 30 seconds at the same resolution as used in the EB imaging.

Metabolic Substrate Assessment: mESCs were cultured in either standard medium, as above (DMEM, high glucose; Invitrogen) or in DMEM with no glucose and no pyruvate (Invitrogen) that was supplemented with either 25 mM glucose or 1 mM pyruvate (levels equivalent to that found in the standard DMEM, high glucose medium). This provided three treatment conditions: control (glucose and pyruvate), pyruvate only or glucose only. Cells were plated as described for the lactate assay. Cells were visually inspected each day to assess for attachment and morphology. After 3 days, cells were counted and used for the formation of EBs. EBs were formed in hanging drops of the same medium in which the ESCs had been cultured. On the second day after EB formation, 8 to 12 EBs were plated into each well of a 6 well plate, continuing in the same medium in which they were originally cultured. EBs were scored for the presence of beating areas on days 7, 8, 9, 10 and 12 after EB formation. No beating areas were observed in any treatment on day 7, so this time point was not included in the analyses presented. After day 12 one well of each treatment was fixed in 4% paraformaldehyde (EMS, Hatfield, PA) in PBS for 15 min then immunolabeled for cardiac troponin T (cTnT) expression. Following blocking with 5% bovine serum albumin (BSA) (Fischer Scientific), 0.1% TritonX-100 (Fischer Scientific); 2% normal goat serum (Chemicon/Millipore), cells were incubated overnight at 4°C with 1∶200 cTnT antibody (Thermo Scientific). After rinsing with PBS, cells were incubated for 2 hours at room temperature with 1∶500 goat anti-mouse secondary antibody (Alexafluor 647) (MolecularProbes/Life Technologies, Carlsbad, CA), rinsed with PBS and covered with mounting medium. mEBs were imaged using a IX71 Olympus fluorescence microscope using a 4X PlanN 0.10 NA lens. For each EB, images of three contiguous but non-overlapping regions were collected.

### Image Analyses

Intensity: Endogenous fluorescence intensity and GFP intensity were assessed using a custom-developed FIJI ImageJ plugin macro “Intensity Macro for Background Subtraction” (http://www.loci.wisc.edu/software). This plugin allows for uniform background subtraction across all images and measurement of fluorescent intensity with respect to the area of the EB determined from the brightfield image and/or the fluorescence image. The level of background subtracted by the plugin is determined by allowing the user to select a region of background in each image, calculating an intensity histogram of the background region, and subsequently subtracting the value that corresponds to a percentage of the background histogram that the user desires to retain. After background subtraction, the user is able to outline the EB from the brightfield image to allow for the plugin to calculate a background-subtracted, fluorescent intensity per pixel value for each image. Intensity of the Oct4 antibody labeling for the forced differentiation experiments was determined manually by setting threshold values to subtract 99% of the background intensity and dividing resulting the total intensity by area of the brightfield image to get the intensity/pixel intensity value. All image analysis was performed on raw image data. For cTnT labeled EBs, because the entire field of view for each region of interest was covered with cells, the intensity was measured in ImageJ by subtracting the secondary only intensity level from each pixel in each data image. The intensity of the entire field of view was then measured to determine mean intensity per pixel. For publication purposes, brightness of images was enhanced in Adobe Photoshop CS5 (Adobe Systems, San Jose, CA) only after assembly into figures and applied equally across images within a data set.

Fluorescence lifetime: Fluorescence lifetime data for global analyses were analyzed using SPCImage (Becker and Hickl) while cell by cell analyses were performed using TRI2 (Gray Institute, Oxford) due to its batch processing capabilities, masking tools and data export options. In SPCImage, an incomplete model with a two component fit was used. Quality of fit was assessed by Chi^2^ values and any sample with an average Chi^2^ value ≥ to 1.5 was not included in the analysis. In TRI2, each lifetime image was fitted to a bi-exponential decay model using the Levenberg-Marquardt method with a “very strict” stopping criterion and a target Chi^2^ value <1.1. The fitting of each image also incorporated the deconvolution of the instrument response. Data were exported as images for each lifetime parameter and imported into FIJI ImageJ along with the corresponding GFP image. Each set of images were verified to be co-registered. Cells that were adherent to other cells (as opposed to rounded and detaching), as a criterion for viability, and with identifiable boundaries were outlined and measured for each lifetime parameter, endogenous intensity and GFP intensity. Raw endogenous intensity and lifetime parameters were normalized by the mean value of the corresponding parameter for each EB. For each parameter, a value <1.0 indicates that for a particular cell, that parameter was less than the mean value for cells in that EB whereas a value >1.0 indicates that parameter was greater than the mean value for cells in that EB.

### Statistical Analyses

Statistical analyses were conducted using SigmaPlot 11.0 (Systat Software, Chicago, IL). *P*<0.05 was the criterion used for considering significance for all analyses. Two group comparisons were performed using t-tests. Data sets that failed the normality test or equal variance test, the non-parametric Mann-Whitney Rank Sum test was utilized. For multiple comparisons, one way analysis of variance was performed, using the Kruskal-Wallis one way analysis of variance on ranks when data sets failed equal variance test, followed by Dunn’s method for the multiple pairwise comparison procedure. For experiments where the same samples were imaged over multiple days, a repeated measures ANOVA was used, followed by the Holm-Sidak method for multiple pairwise comparisons. Pearson’s correlation coefficients were tabulated to determine correlations between GFP intensity (corresponding to Oct4 expression) and endogenous fluorescence parameters. Differences in proportions of EBs with beating areas were assessed using Chi-square analysis.

## Supporting Information

Figure S1 **Additional analysis of global endogenous fluorescence intensity of NADH with development of EBs, associated with** **Figure 1** **.** A. Complete time course of MPLSM single optical sections of mESCs at different stages of differentiation (ESC and days 1, 2, 3, 2, 5, and 8 of EB formation) using 780 nm excitation. Top row shows raw data while the bottom row shows images following application of a background subtraction FIJI plugin macro (see Methods) to remove 90% of the background prior to intensity analysis of the region of interest. Scale bar = 50 µm. B. Details of statistical comparison of normalized intensities of EBs compared to the ESCs imaged on the same day. *P* values in red indicate statistical difference (*P*<0.05) between EB and corresponding ESC intensity. C. Details of t-test comparisons of normalized EBs on each day with EBs of every other day. *P* values in red indicate statistical difference (*P*<0.05) between days; t-test. * indicates difference at *P*<0.05, as determined by Dunn’s Method for pairwise multiple comparison procedures following Kruskal-Wallis one way ANOVA on ranks.
(TIF)

Figure S2 **Additional analysis of changes in NADH endogenous fluorescence lifetime with mESC developmental age, associated with** **Figure 1** **.** A. Color mapped lifetime values on complete time course of MPLSM single optical sections of mouse ESCs at different stages of differentiation (mESC and days 1, 2, 3, 4, 5, and 8 of EB formation) using 780 nm excitation. Color bar indicates lifetime values for either τ_m_ (top row) or τ_2_ (bound NADH; bottom row). Scale bar = 50 µm. Bar graphs for normalized τ_m_ (B - in Figure 1) or τ_2_ (C) including bars for ESC data. Tables showing t-test comparisons of each normalized lifetime values on a given day to every other day for τ_m_ (D) and τ_2_ (E). *P*-values in red indicate statistical difference (*P* = 0.05), t-test; * indicates difference at *P*<0.05, as determined by Dunn’s Method for pairwise multiple comparison procedures following Kruskal-Wallis one way ANOVA on ranks. Quantitation of lifetime values for τ_1_ (F) and α_2_ (G). For this and subsequent figures, α_1_ comparisons are not shown because α_1_ is linearly dependent on α_2_ in the relationship α_1_+ α_2_ = 1. Values are normalized to corresponding mean mESC lifetime values. * indicates statistical difference (P<0.05; t-test) between mEB and corresponding mESC lifetimes for that day while horizontal lines indicate statistical difference between normalized mEB lifetime values (*P*<0.05, Dunn’s Method for pairwise multiple comparison procedures following Kruskal-Wallis one way ANOVA on ranks). Tables showing t-test comparisons of each normalized lifetime values on a given day to every other day for τ_1_ (H) and α_2_ (I). *P*-values in red indicate statistical difference (*P* = 0.05); * indicates difference at *P*<0.05, as determined by Dunn’s Method for pairwise multiple comparison procedures following Kruskal-Wallis one way ANOVA on ranks.
(TIF)

Figure S3 **Endogenous fluorescence intensity and lifetime in mESCs driven to differentiate.** A. Background subtracted intensity of MPLSM single optical sections of live mESCs at 0, 3, and 6 days of RA treatment using 780 nm excitation. B. Quantitation of background subtracted endogenous fluorescence. C. Color mapped mean lifetime (τ_m_) images of endogenous fluorescence, with quantitation in D, of same regions as in A. E. Oct4 antibody immunofluorescent label in fixed samples corresponding to live endogenous fluorescence images in A and C. Images are a sum of multiple MPLSM optical sections. F. Quantitation of Oct4 immunofluorescence as fluorescence intensity per nucleus. G. Same cells showing DAPI labeled nuclei. Brightfield images of the same region of cells as presented in A, prior to (H) and after (I) fixation to show the changes that occur following fixation. Scale bar = 50 µm. Quantitation of additional lifetime parameters, τ_1_(J), τ_2_ (K) and α_2_ (L). * indicates difference from Day 0 (*P*<0.05, Mann-Whitney).
(TIF)

Figure S4 **Spectral separation of GFP and endogenous fluorescence.** Extrinsically fluorescent Oct4-GFP mESC and non-fluorescent HM1 ESC colonies were imaged using MPLSM at 890 nm to excite GFP and at 780 nm to excite NADH while bandpass filters were used to segregate emission spectral for NADH (457/50 nm) and GFP (520/35 nm). Combinations of excitation wavelength and filters are shown as “excitation wavelength/peak emission wavelength” (ex/em) in nm. A. Oct4-GFP expressing mESCs imaged with the various ex/em combinations. GFP does not spectrally overlap into the NADH filter range (ex 890/em 457) and NADH fluorescence is not induced at 890 nm (ex 890/em 457). (B) Non-GFP expressing cells imaged to assess the basal level of autofluorescence 890 nm and imaged with the various ex/em combinations. Minimal autofluorescence is detected with 890 nm excitation (ex 890/em 520). Furthermore, the metabolic coenzyme FAD, the main contributor of intrinsic fluorescence at 890nm, exhibits minimal spectral overlap with the NADH emission filter range (ex 890/em 457). Scale bar = 50 µm.
(TIF)

Figure S5 **Quantitation of additional lifetime parameters for mEB cellular analysis, associated with** **Figure 3** **.** For the Oct4-GFP mESC experiments and cellular analysis shown in Figure 3, the lifetime parameters τ_1_, and α2 were also quantified. The same ROIs used for Figure 2 were evaluated for these parameters and classified into the same categories of GFP(H), GFP(M) and GFP(L). A. Graph of the parameter τ_1_, the short lifetime component (free NADH). B. Graph showing the lifetime parameter α_2_, corresponding to the proportional contribution of the long lifetime component (bound NADH). * indicates statistical difference from GFP(H), *P*<0.05 Dunn’s method pair wise comparison following Kruskal-Wallis one way ANOVA on ranks.
(TIF)

Figure S6 **Additional quantitation of global hEB endogenous fluorescence time course, associate with** **Figure 4** **.** Details of statistical comparisons of endogenous fluorescence parameters of hEBs over time shown in graphs in Figure 4. A. Endogenous fluorescence intensity B. τ_m_. Graphs showing quantitation of additional endogenous fluorescence parameters τ_1_ (C), τ_2_ (D), α_2_ (E). F. Details of statistical comparisons for τ_2_. P values in red indicate statistical difference (*P*<0.05) between days using Holm-Sidak method for multiple comparisons following one way repeated measures ANOVA.
(TIF)

Figure S7 **Quantitation of additional lifetime parameters of hEB cellular analysis associated with** **Figure 5** **.** Along with the τ_m_ and τ_2_ shown in Figure 5, the lifetime parameters τ_1_ and α_2_ were quantified. The same ROIs used in Figure 5 were evaluated and classified into the two categories of GFP(H) and GFP(L). A. Graph of the parameter τ_1_, the short lifetime component (free NADH). B. Graph showing the lifetime parameter α_2_, corresponding to the proportional contribution of the long lifetime component (bound NADH). * indicates statistical difference (*P*<0.05) from GFP(H): for τ_1_ *P* = 0.046 (Mann-Whitney), for α_2_ *P* = 0.071.
(TIF)
