# Supplementary figures and images for: Endogenous Fluorescence Signatures in Living Pluripotent Stem Cells Change with Loss of Potency
Source: PLoS One. 2012 Aug 29;7(8):e43708. doi: 10.1371/journal.pone.0043708 (PMC3430704; doi:10.1371/journal.pone.0043708)

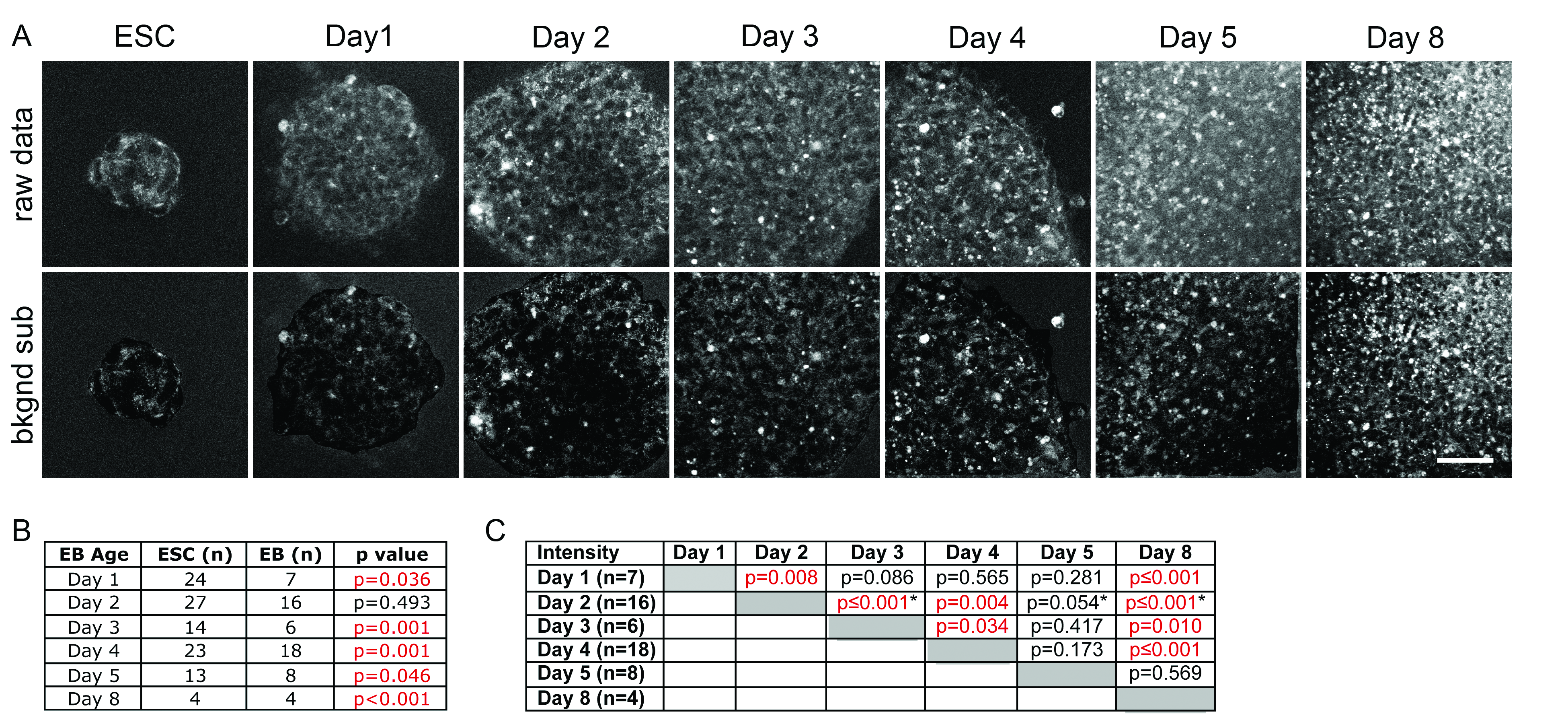

Supplement: Figure S1 — Additional analysis of global endogenous fluorescence intensity of NADH with development of EBs, associated with Figure 1 . A. Complete time course of MPLSM single optical sections of mESCs at different stages of differentiation (ESC and days 1, 2, 3, 2, 5, and 8 of EB formation) using 780 nm excitation. Top row shows raw data while the bottom row shows images following application of a background subtraction FIJI plugin macro (see Methods) to remove 90% of the background prior to intensity analysis of the region of interest. Scale bar = 50 µm. B. Details of statistical comparison of normalized intensities of EBs compared to the ESCs imaged on the same day. P values in red indicate statistical difference (P<0.05) between EB and corresponding ESC intensity. C. Details of t-test comparisons of normalized EBs on each day with EBs of every other day. P values in red indicate statistical difference (P<0.05) between days; t-test. * indicates difference at P<0.05, as determined by Dunn’s Method for pairwise multiple comparison procedures following Kruskal-Wallis one way ANOVA on ranks. (TIF) [file pone.0043708.s001.tif]

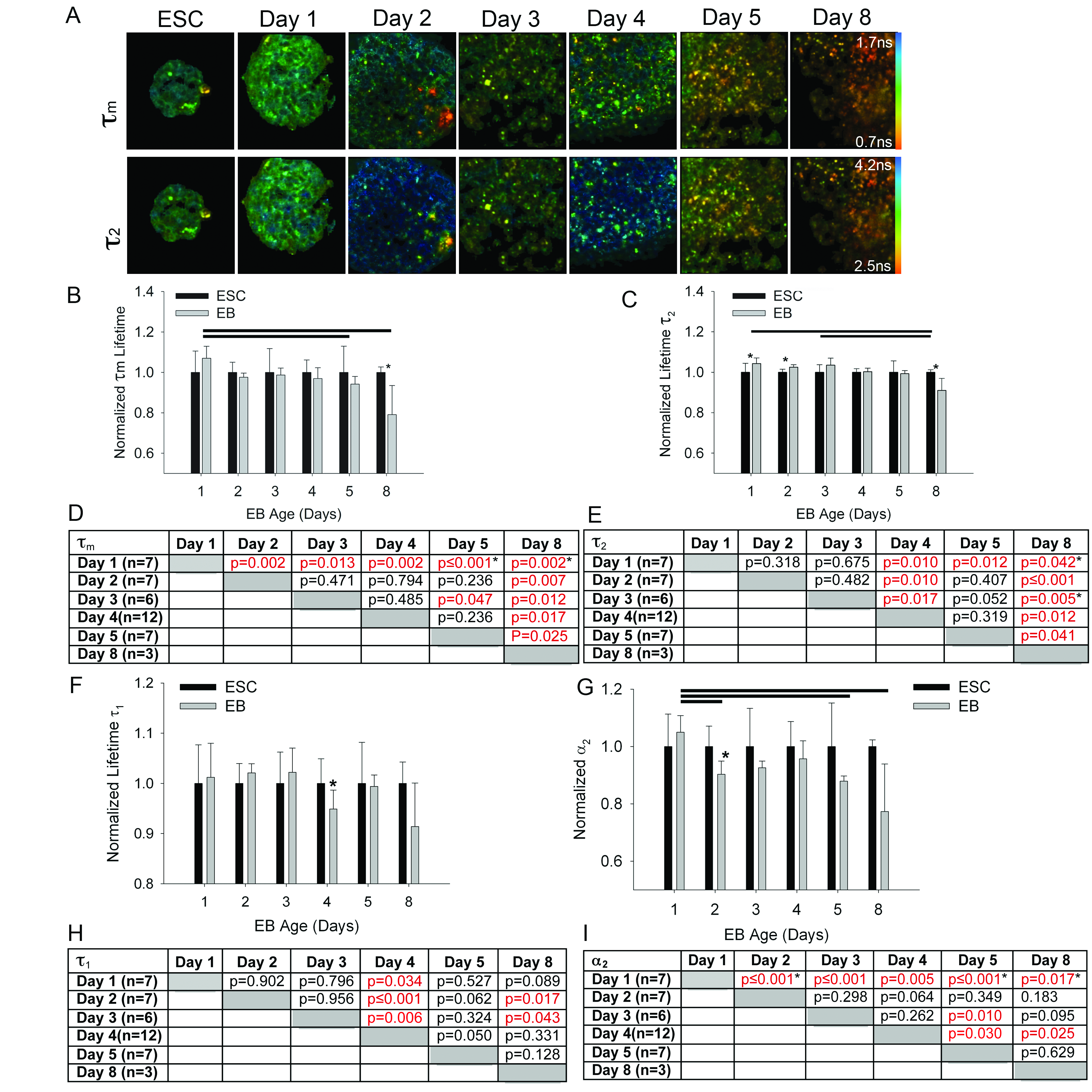

Supplement: Figure S2 — Additional analysis of changes in NADH endogenous fluorescence lifetime with mESC developmental age, associated with Figure 1 . A. Color mapped lifetime values on complete time course of MPLSM single optical sections of mouse ESCs at different stages of differentiation (mESC and days 1, 2, 3, 4, 5, and 8 of EB formation) using 780 nm excitation. Color bar indicates lifetime values for either τm (top row) or τ2 (bound NADH; bottom row). Scale bar = 50 µm. Bar graphs for normalized τm (B - in Figure 1) or τ2 (C) including bars for ESC data. Tables showing t-test comparisons of each normalized lifetime values on a given day to every other day for τm (D) and τ2 (E). P-values in red indicate statistical difference (P = 0.05), t-test; * indicates difference at P<0.05, as determined by Dunn’s Method for pairwise multiple comparison procedures following Kruskal-Wallis one way ANOVA on ranks. Quantitation of lifetime values for τ1 (F) and α2 (G). For this and subsequent figures, α1 comparisons are not shown because α1 is linearly dependent on α2 in the relationship α1+ α2 = 1. Values are normalized to corresponding mean mESC lifetime values. * indicates statistical difference (P<0.05; t-test) between mEB and corresponding mESC lifetimes for that day while horizontal lines indicate statistical difference between normalized mEB lifetime values (P<0.05, Dunn’s Method for pairwise multiple comparison procedures following Kruskal-Wallis one way ANOVA on ranks). Tables showing t-test comparisons of each normalized lifetime values on a given day to every other day for τ1 (H) and α2 (I). P-values in red indicate statistical difference (P = 0.05); * indicates difference at P<0.05, as determined by Dunn’s Method for pairwise multiple comparison procedures following Kruskal-Wallis one way ANOVA on ranks. (TIF) [file pone.0043708.s002.tif]

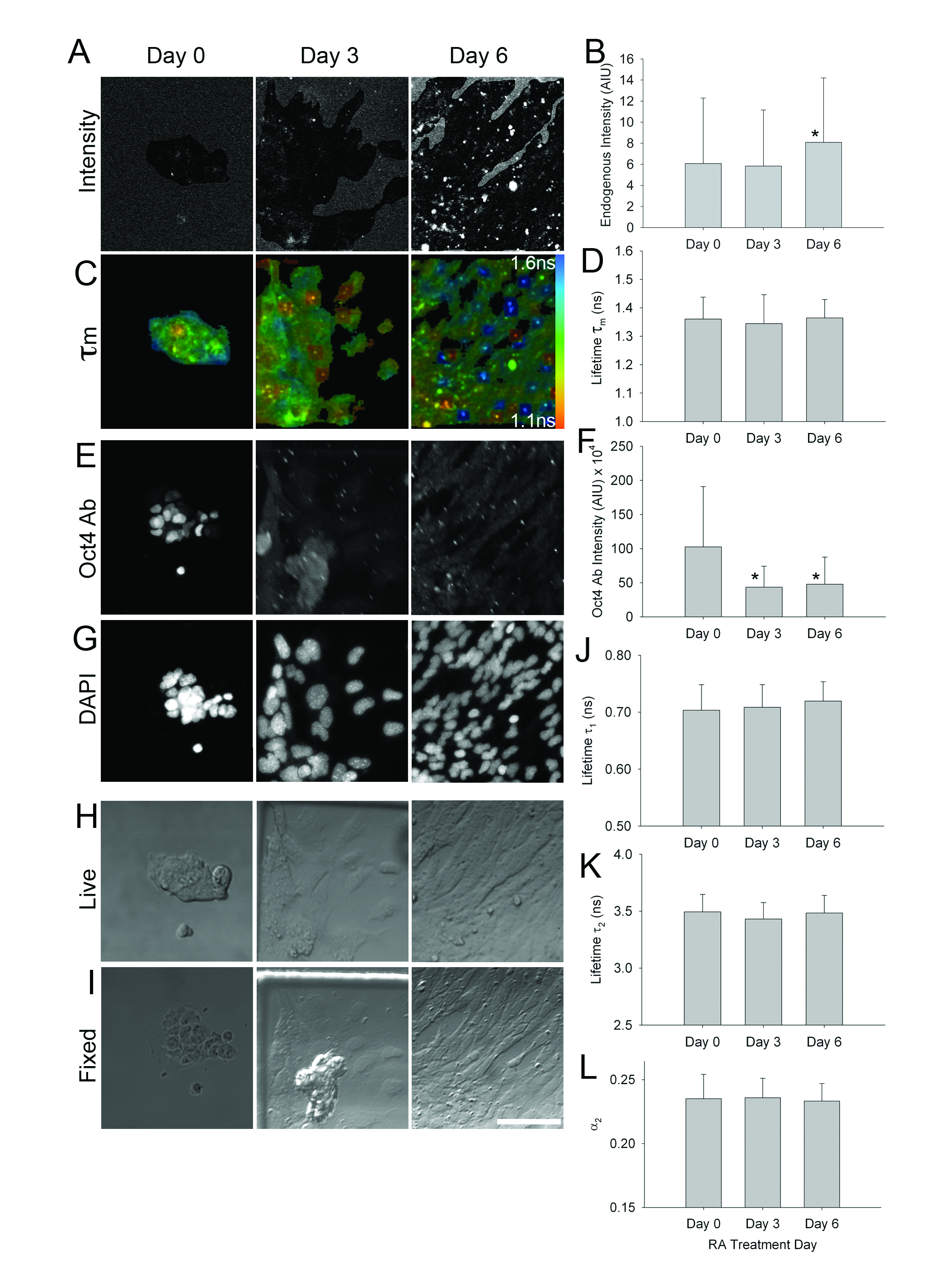

Supplement: Figure S3 — Endogenous fluorescence intensity and lifetime in mESCs driven to differentiate. A. Background subtracted intensity of MPLSM single optical sections of live mESCs at 0, 3, and 6 days of RA treatment using 780 nm excitation. B. Quantitation of background subtracted endogenous fluorescence. C. Color mapped mean lifetime (τm) images of endogenous fluorescence, with quantitation in D, of same regions as in A. E. Oct4 antibody immunofluorescent label in fixed samples corresponding to live endogenous fluorescence images in A and C. Images are a sum of multiple MPLSM optical sections. F. Quantitation of Oct4 immunofluorescence as fluorescence intensity per nucleus. G. Same cells showing DAPI labeled nuclei. Brightfield images of the same region of cells as presented in A, prior to (H) and after (I) fixation to show the changes that occur following fixation. Scale bar = 50 µm. Quantitation of additional lifetime parameters, τ1(J), τ2 (K) and α2 (L). * indicates difference from Day 0 (P<0.05, Mann-Whitney). (TIF) [file pone.0043708.s003.tif]

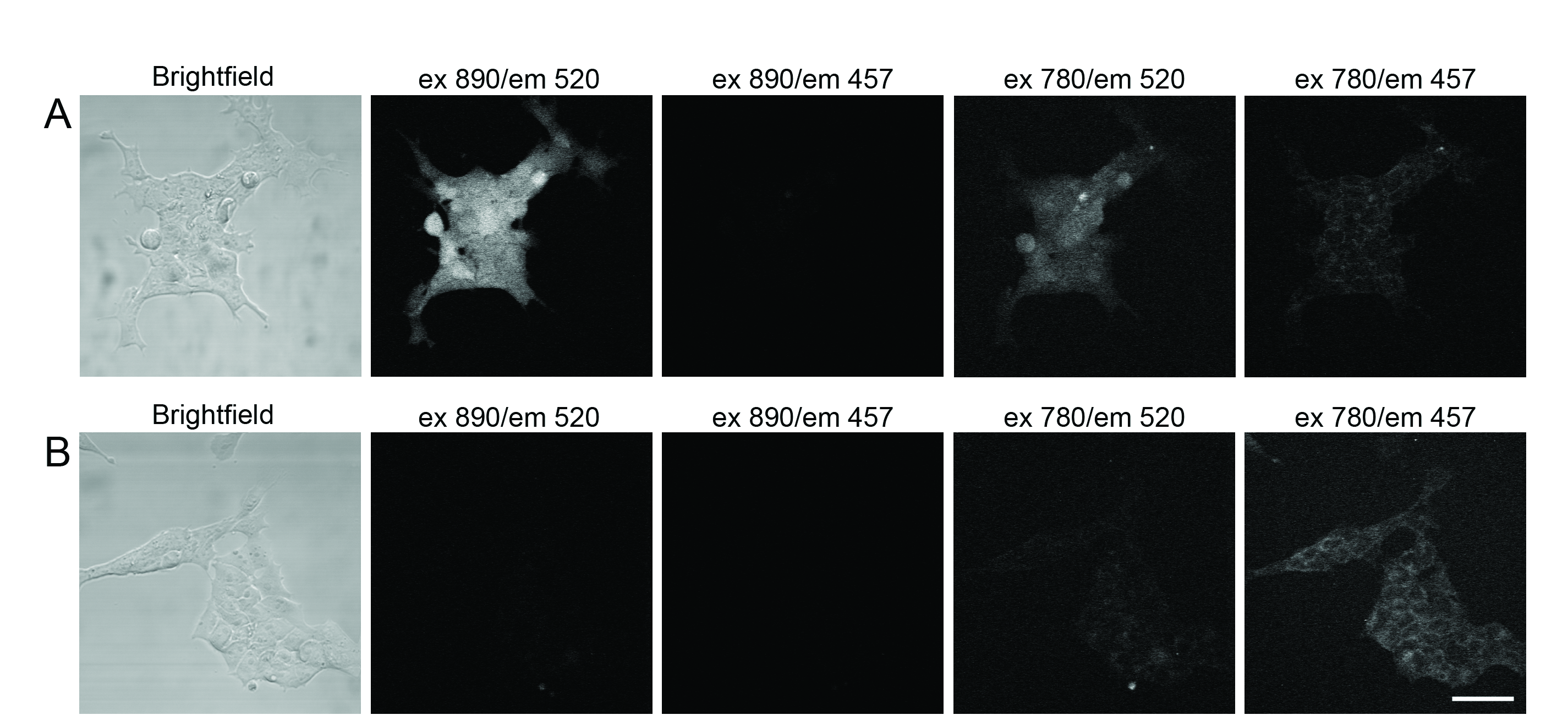

Supplement: Figure S4 — Spectral separation of GFP and endogenous fluorescence. Extrinsically fluorescent Oct4-GFP mESC and non-fluorescent HM1 ESC colonies were imaged using MPLSM at 890 nm to excite GFP and at 780 nm to excite NADH while bandpass filters were used to segregate emission spectral for NADH (457/50 nm) and GFP (520/35 nm). Combinations of excitation wavelength and filters are shown as “excitation wavelength/peak emission wavelength” (ex/em) in nm. A. Oct4-GFP expressing mESCs imaged with the various ex/em combinations. GFP does not spectrally overlap into the NADH filter range (ex 890/em 457) and NADH fluorescence is not induced at 890 nm (ex 890/em 457). (B) Non-GFP expressing cells imaged to assess the basal level of autofluorescence 890 nm and imaged with the various ex/em combinations. Minimal autofluorescence is detected with 890 nm excitation (ex 890/em 520). Furthermore, the metabolic coenzyme FAD, the main contributor of intrinsic fluorescence at 890nm, exhibits minimal spectral overlap with the NADH emission filter range (ex 890/em 457). Scale bar = 50 µm. (TIF) [file pone.0043708.s004.tif]

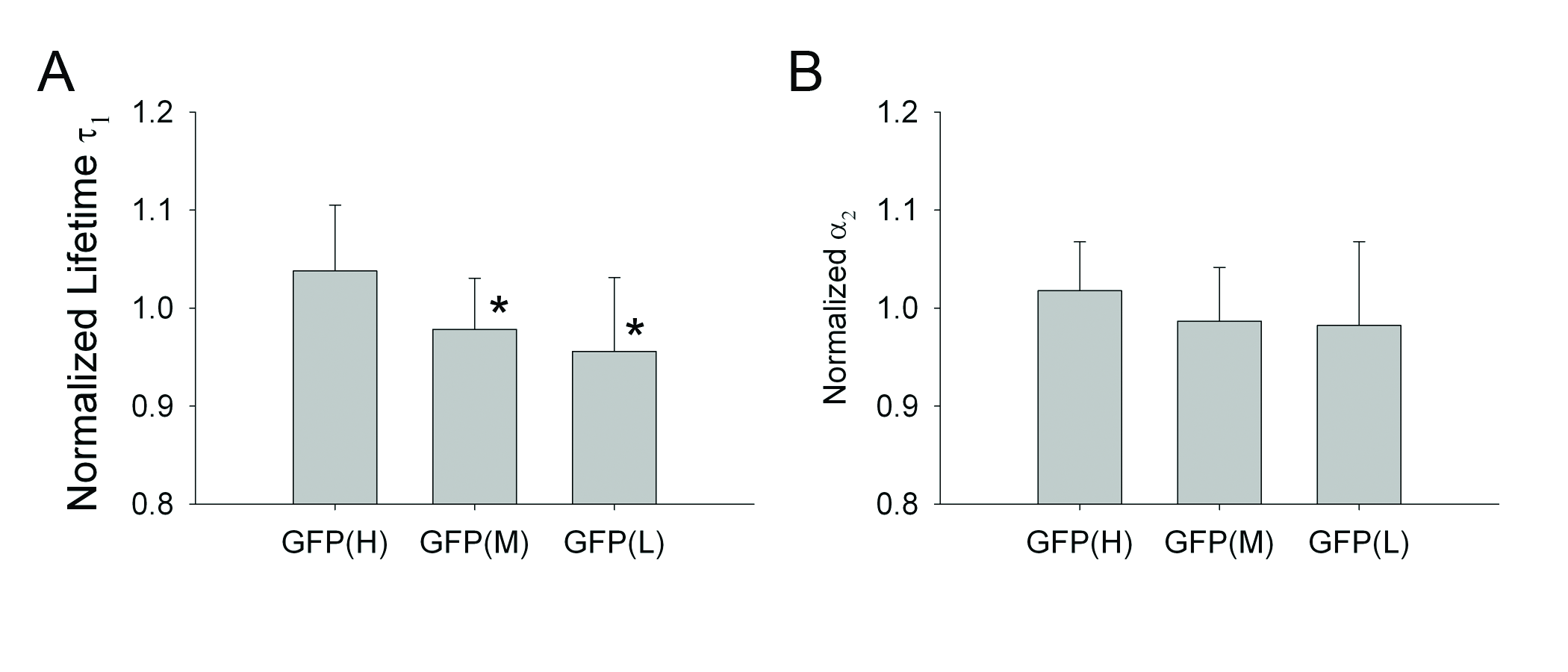

Supplement: Figure S5 — Quantitation of additional lifetime parameters for mEB cellular analysis, associated with Figure 3 . For the Oct4-GFP mESC experiments and cellular analysis shown in Figure 3, the lifetime parameters τ1, and α2 were also quantified. The same ROIs used for Figure 2 were evaluated for these parameters and classified into the same categories of GFP(H), GFP(M) and GFP(L). A. Graph of the parameter τ1, the short lifetime component (free NADH). B. Graph showing the lifetime parameter α2, corresponding to the proportional contribution of the long lifetime component (bound NADH). * indicates statistical difference from GFP(H), P<0.05 Dunn’s method pair wise comparison following Kruskal-Wallis one way ANOVA on ranks. (TIF) [file pone.0043708.s005.tif]

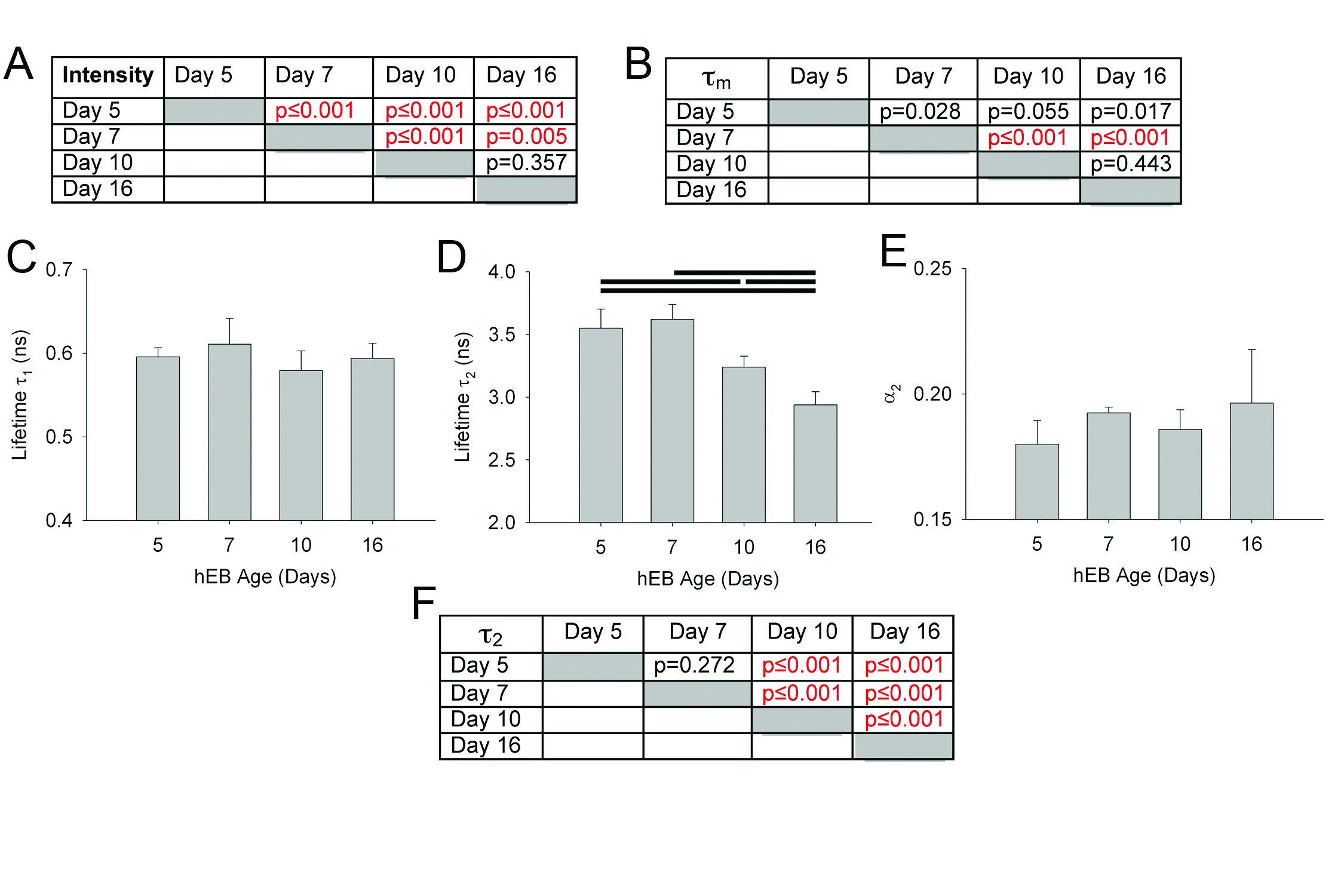

Supplement: Figure S6 — Additional quantitation of global hEB endogenous fluorescence time course, associate with Figure 4 . Details of statistical comparisons of endogenous fluorescence parameters of hEBs over time shown in graphs in Figure 4. A. Endogenous fluorescence intensity B. τm. Graphs showing quantitation of additional endogenous fluorescence parameters τ1 (C), τ2 (D), α2 (E). F. Details of statistical comparisons for τ2. P values in red indicate statistical difference (P<0.05) between days using Holm-Sidak method for multiple comparisons following one way repeated measures ANOVA. (TIF) [file pone.0043708.s006.tif]

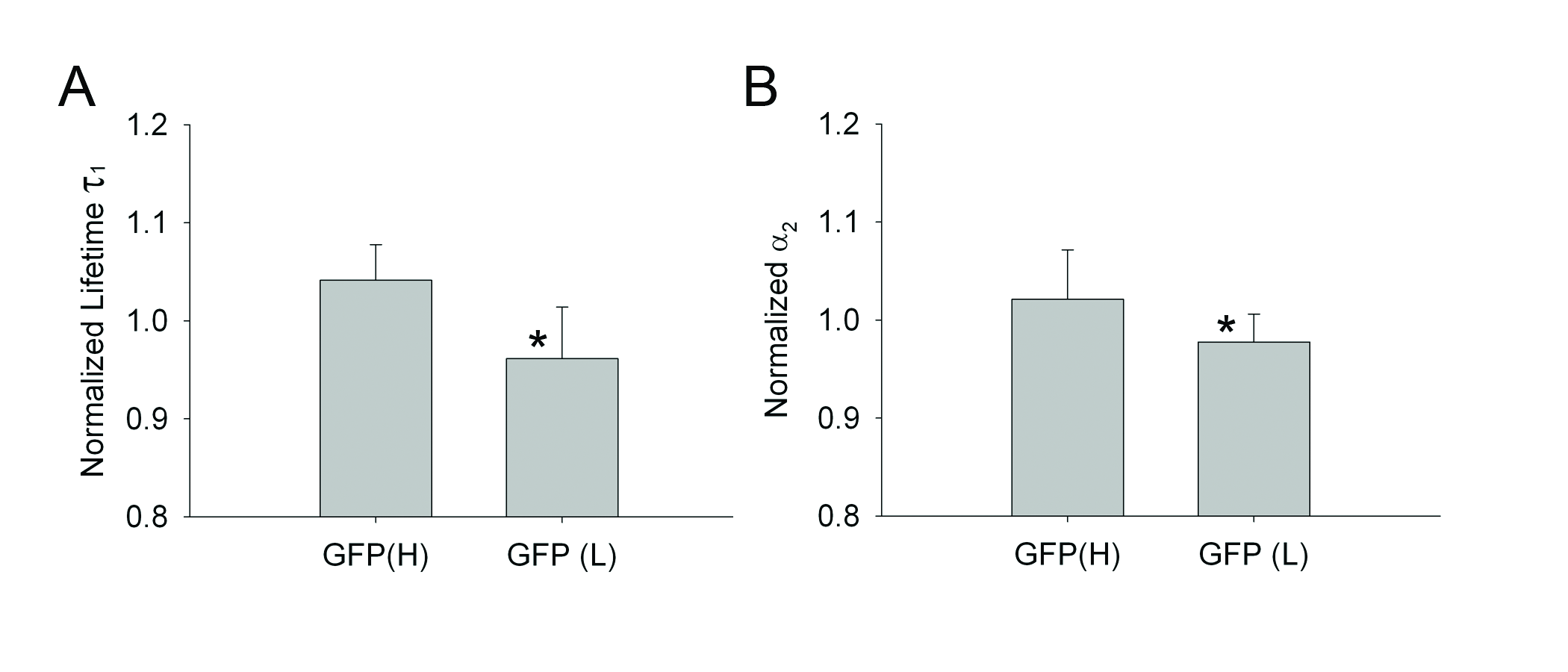

Supplement: Figure S7 — Quantitation of additional lifetime parameters of hEB cellular analysis associated with Figure 5 . Along with the τm and τ2 shown in Figure 5, the lifetime parameters τ1 and α2 were quantified. The same ROIs used in Figure 5 were evaluated and classified into the two categories of GFP(H) and GFP(L). A. Graph of the parameter τ1, the short lifetime component (free NADH). B. Graph showing the lifetime parameter α2, corresponding to the proportional contribution of the long lifetime component (bound NADH). * indicates statistical difference (P<0.05) from GFP(H): for τ1 P = 0.046 (Mann-Whitney), for α2 P = 0.071. (TIF) [file pone.0043708.s007.tif]
